# Gut epithelial electrical cues drive differential localization of enterobacteria

**DOI:** 10.1038/s41564-024-01778-8

**Published:** 2024-08-20

**Authors:** Yaohui Sun, Fernando Ferreira, Brian Reid, Kan Zhu, Li Ma, Briana M. Young, Catherine E. Hagan, Renée M. Tsolis, Alex Mogilner, Min Zhao

**Affiliations:** 1 Department of Ophthalmology and Vision Science, Department of Dermatology, School of Medicine, University of California, Davis, Sacramento, CA, USA.; 2 Department of Internal Medicine, School of Medicine, University of California, Davis, Sacramento, CA, USA.; 3 Departamento de Biologia, Centro de Biologia Molecular e Ambiental (CBMA), Universidade do Minho, Braga, Portugal.; 4 Department of Medical Microbiology and Immunology, School of Medicine, University of California, Davis, Davis, CA, USA.; 5 Department of Comparative Medicine, School of Medicine, University of Washington, Seattle, WA, USA.; 6 Courant Institute and Department of Biology, New York University, New York, NY, USA.; 7 Present address: Coty R&D Technology and Innovation, Shanghai, P. R. China.

## Abstract

*Salmonella* translocate to the gut epithelium via microfold cells lining the follicle-associated epithelium (FAE). How *Salmonella* localize to the FAE is not well characterized. Here we use live imaging and competitive assays between wild-type and chemotaxis-deficient mutants to show that *Salmonella enterica* serotype Typhimurium (*S*. Typhimurium) localize to the FAE independently of chemotaxis in an ex vivo mouse caecum infection model. Electrical recordings revealed polarized FAE with sustained outward current and small transepithelial potential, while the surrounding villus is depolarized with inward current and large transepithelial potential. The distinct electrical potentials attracted *S*. Typhimurium to the FAE while *Escherichia coli* (*E. coli*) localized to the villi, through a process called galvanotaxis. Chloride flux involving the cystic fibrosis transmembrane conductance regulator (CFTR) generated the ionic currents around the FAE. Pharmacological inhibition of CFTR decreased *S*. Typhimurium FAE localization but increased *E. coli* recruitment. Altogether, our findings demonstrate that bioelectric cues contribute to *S*. Typhimurium targeting of specific gut epithelial locations, with potential implications for other enteric bacterial infections.

Our gut contains ~100 trillion commensal bacteria that aid in nutrient absorption and immune maturation, and protect the host from bacterial infections^1^. Despite this, many enteric pathogens, such as *Salmonella*, *Shigella*, *Yersinia* and pathogenic *Escherichia coli* (*E. coli*), have developed strategies to colonize the intestinal mucosa and cause diseases. These pathogens are a major public health concern due to their ability to cause severe diarrhoeal and extraintestinal diseases and their ease of transmission through contaminated food and water^2–4^. *Salmonella* and other enteric pathogens use a type III secretion system^5^ to invade host cells, targeting a small number of follicle-associated epithelial (FAE) cells known as microfold (M) cells^6–8^. Contamination with *Salmonella*, even in small quantities, can cause severe enteritis and/or disseminated infections^9,10^. However, it is not well understood how a small load of pathogens navigate to the vulnerable FAE entry point amid millions or billions of commensal microbes^11^.

Bioelectricity, foundational in modern electrophysiology, has been demonstrated across various species and tissues, from *Dictyostelium*^12^ to mammals^13,14^, and from neuronal^15,16^ to epithelial tissues^17,18^. Live cells maintain a transmembrane potential (*V*_m_) crucial for many cellular functions^19^. Polarized cell sheets, such as epithelia and endothelia, generate electrical potentials from asymmetrically organized channels and pumps with roles in cell migration and wound healing^20,21^. The cystic fibrosis transmembrane conductance regulator (CFTR) maintains the transepithelial potential across epithelial tissues by enabling chloride secretion and supporting bicarbonate transport, critical for optimal epithelial function^22^. CFTR dysfunction, as seen in cystic fibrosis, disrupts ion transport and transepithelial potential (TEP), leading to gastrointestinal problems^23^.

The intestinal epithelial landscape shows morphological and functional differences between villus epithelium and FAE. Villus epithelium is composed of enterocytes with microvilli for nutrient absorption, whereas FAE contains M cells overlying Peyer’s patches for antigen sampling^24^. Using a mouse caecum model, we discovered that *Salmonella* infection-generated electric fields in gut epithelia contribute to systemic bacterial infections. We observed differences in TEPs between FAE and surrounding villus epithelium^25^. However, it is unknown how these regional bioelectric activities are generated and organized, and how their configuration contributes to pathogenic bacterial targeting. *Salmonella enterica* serotype Typhimurium (*S*. Typhimurium) and commensal *E. coli* with no or reduced O-antigen^26^ have distinct surface electrical properties and respond differently to electrical fields^27^. This distinct response is determined by the disparity in passive electrophoretic mobilities of their cell body versus flagellar filaments^28^. We hypothesized that enteric pathogens such as *S*. Typhimurium use a galvanotactic mechanism to target invasion sites. To test this, we found that *S*. Typhimurium targets FAE invasively by exploiting a local, sustained bioelectric network in the gut epithelia, while commensal *E. coli* avoids the FAE. This process is independent of chemotaxis as chemotaxis-deficient *cheB Salmonella* mutants still undergo galvanotaxis and are attracted to the FAE. These findings have implications for enterobacterial pathogenesis and research on mucous epithelia.

## Results

### *S*. Typhimurium localizes at FAE in an ex vivo caecum model

**Extended Data Fig. 1 |:**
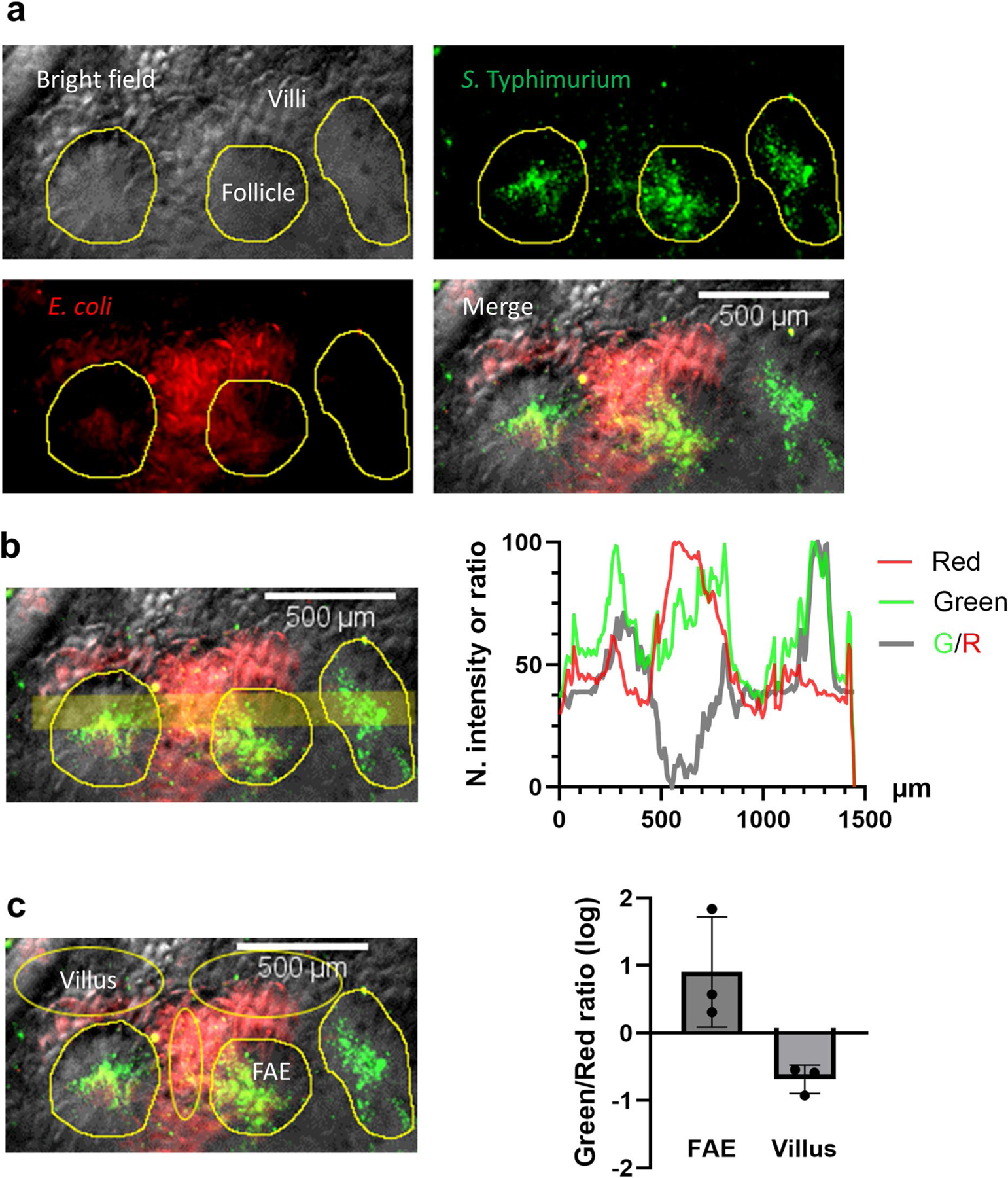
Defining spatial distributions of *S*. Typhimurium and *E. coli* between functionally different FAE and villus epithelium in an *exvivo* mouse caecum model. **a**, Representative images of the mucosal epithelium of a mouse caecum shows the organization of FAE and villi and accumulations of *S*. Typhimurium tagged with EGFP and *E. coli* tagged with dTomato. Pictures were taken at 30 min after incubation. Follicles are outlined in bright field image (yellow enclosures) and transferred to the green and red fluorescent images taken from the same field. Bar, 500 μm. **b**, Spatial profiles of the green vs red signals were calculated via line scan (thick yellow line) and plotted as normalized mean fluorescent intensities or green/red ratio crossing multiple FAEs. **c**, Ratio of mean green vs. red fluorescent intensities of the outlined FAEs (n = 3) were compared to that of matched villus regions (n = 3, yellow eclipses) and plotted as log10 (Mean ± SEM) in a bar chart. This figure is for demonstration purpose and is not subject to a statistical analysis.

**Fig. 1 |:**
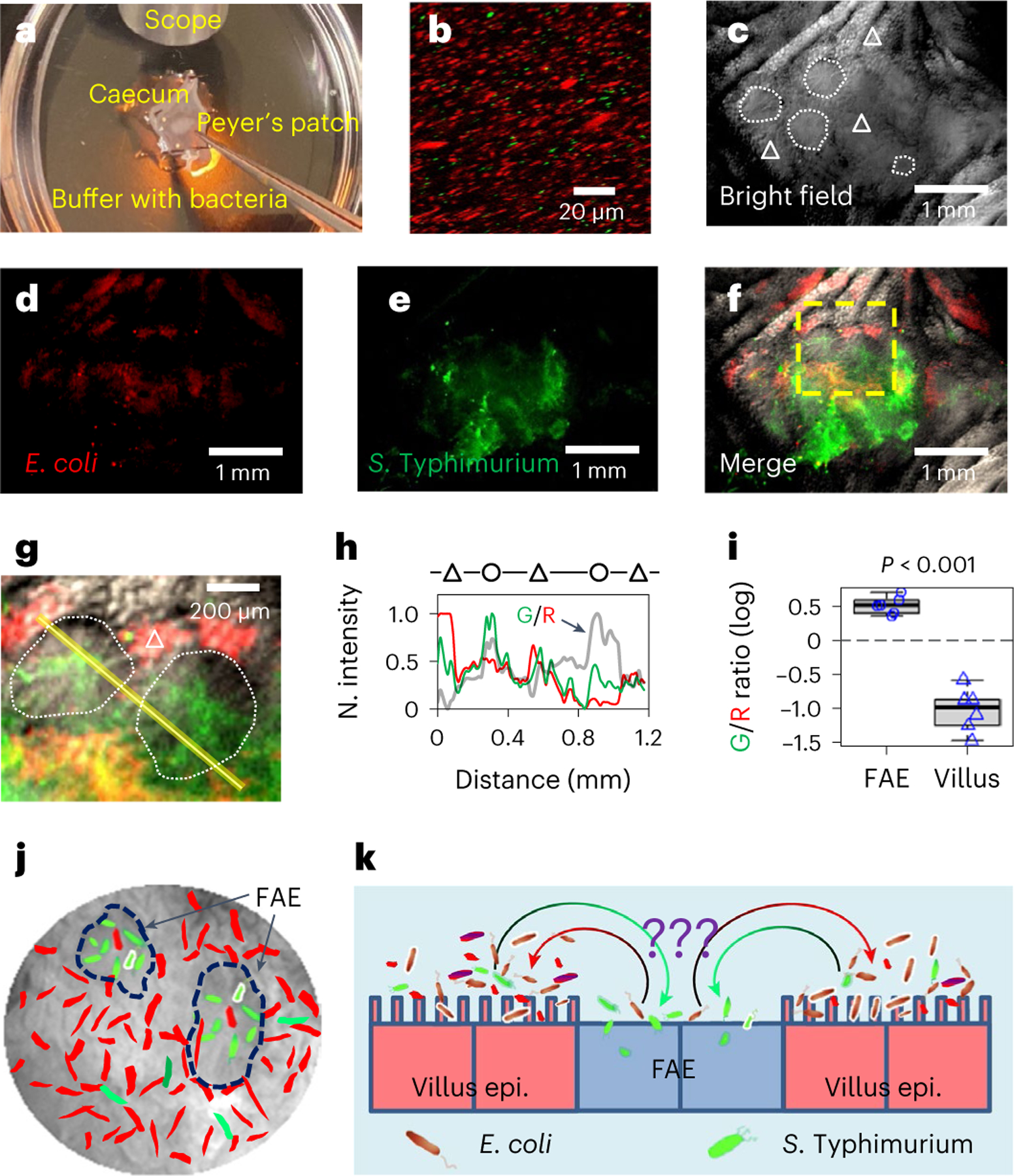
*S*. Typhimurium amasses in FAE and *E. coli* avoids the FAE. **a**, Schematic illustrating the *S*. Typhimurium (expressing EGFP) vs *E. coli* (expressing dTomato) competitive targeting experiment setup in an ex vivo mouse caecum model. A freshly isolated mouse caecum was mounted in a silicone gel plate with its luminal side facing up. Tweezers point to a Peyer’s patch (details in Methods). **b**, A confocal image shows the inoculum of *E. coli* (red) vs *S*. Typhimurium (green) mixture (20:1, 10^8^ c.f.u.s ml^−1^ in mouse Ringer’s solution). **c**–**f**, Bright-field images of the mucosal epithelium of a mouse caecum shows the organization of FAE (white dotted enclosure) and villi (white triangle) (**c**), RFP fluorescence image of *E. coli* expressing dTomato (**d**), GFP fluorescence image of *S*. Typhimurium expressing EGFP (**e**) and the overlay (**f**). **g**, Enlargement of the yellow dashed area in **f**, showing that *S*. Typhimurium (green) preferably colonized FAE (white dotted enclosure), while *E. coli* (red) are dominantly associated with villus epithelium (white triangle). **h**, Normalized fluorescence profiles and green/red fluorescence ratio (thick grey line indicated by an arrow) of the line scan in **g**, showing difference in *S*. Typhimurium (green) and *E. coli* (red) spatial distributions between FAE (circle) and inter- and extrafollicular villus epithelium (triangle). **i**, Mean green/red fluorescence intensity (G/R) ratios associated with FAE or villus epithelium plotted in common logarithm (*n* = 6 mice, *P* < 0.001 by unpaired, two-tailed Student’s *t*-test). Box tops indicate the 75th percentile, box bottoms indicate the 25th percentile, centre lines indicate median, and whiskers indicate maximum and minimum. Dashed line indicates the ratio of 1. **j**, Cartoon showing microscopic view with two highlighted FAEs (dashed enclosures); *S*. Typhimurium in green and *E. coli* in red. **k**, Summary of the finding that *S*. Typhimurium (green) navigates to and accumulates in FAE, which *E. coli* (red) avoids and stays away from, through an unknown sorting mechanism (question marks).

It is well established from animal studies that enteric pathogens prefer the FAE as a gateway to invade the host and cause infections^6–8^. This is difficult to replicate in vitro, even with organotypic cultures that mimic some in vivo electrophysiological features^29^. We use our recently developed ex vivo mouse caecum model^25^ (Fig. 1a) to test whether differently tagged *E. coli* (derived from K12) and *S*. Typhimurium (derived from virulent 14028S) (Fig. 1b and Supplementary Table 1) show preferential targeting in the caecal epithelia. *E. coli* tagged with dTomato preferred the villi and avoided the FAEs (Fig. 1c–f and Extended Data Fig. 1a), while *S*. Typhimurium tagged with EGFP showed a preference for the FAEs, where they amassed (Fig. 1c–f and Extended Data Fig. 1a). These different tropisms were confirmed by quantifying the spatial fluorescence intensity profiles (Fig. 1g,h and Extended Data Fig. 1b). Merging channels and calculating spatial *Salmonella* vs *E. coli* ratios showed exclusive colonization of *Salmonella* in the FAEs (*P* < 0.001) (Fig. 1i and Extended Data Fig. 1c). Since targeting FAE is common among enteric pathogens, these data suggest a specific ‘sorting’ mechanism aiding *S*. Typhimurium targeting (Fig. 1j,k).

### Active ionic currents loop between FAE and villus epithelium

**Extended Data Fig. 2 |:**
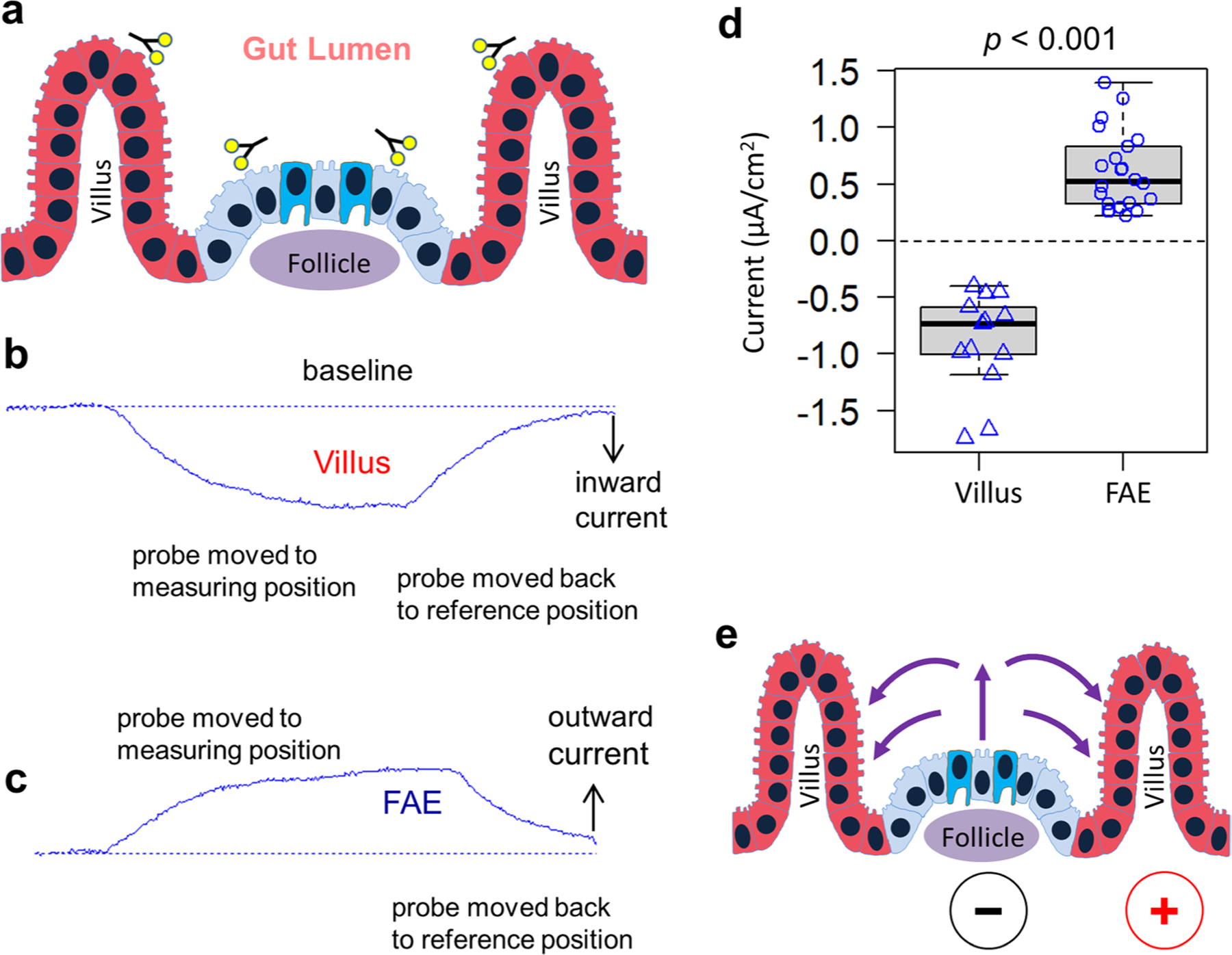
Ionic currents loop and contribute to the establishment of a lateral electrical field between functionally different FAE and villus epithelia. **a**, Schematic depicting the organization of follicles and villi. Forks indicate the sites where the ionic currents were measured using vibrating probes. **b**, A typical current trace recorded at villus epithelium and **c**, A typical current trace recorded at FAE. These traces illustrate how an ionic current is measured and how the current polarities are defined. Maximal ionic currents were calculated based on a standard curve that was generated by passing specific currents in the same experimental setup for each probe. **d**, Consistency of the pooled measurements from different animals and different experiments (n = 13 and 22 mice respectively) including the ones we previously reported^25^, showing that the ionic currents at FAE are exclusively outward and the ionic currents at villi are exclusively inward (*p* < 0.001 by unpaired, two-tailed Student’s *t*-test). Box tops indicate the 75th percentile, box bottoms indicate the 25th percentile, centre lines indicate median, and whiskers indicate maximum and minimum. **e**, A cartoon depicts ionic flow (purple arrows) and the establishment of a lateral electrical field between FAE and villus epithelia.

**Fig. 2 |:**
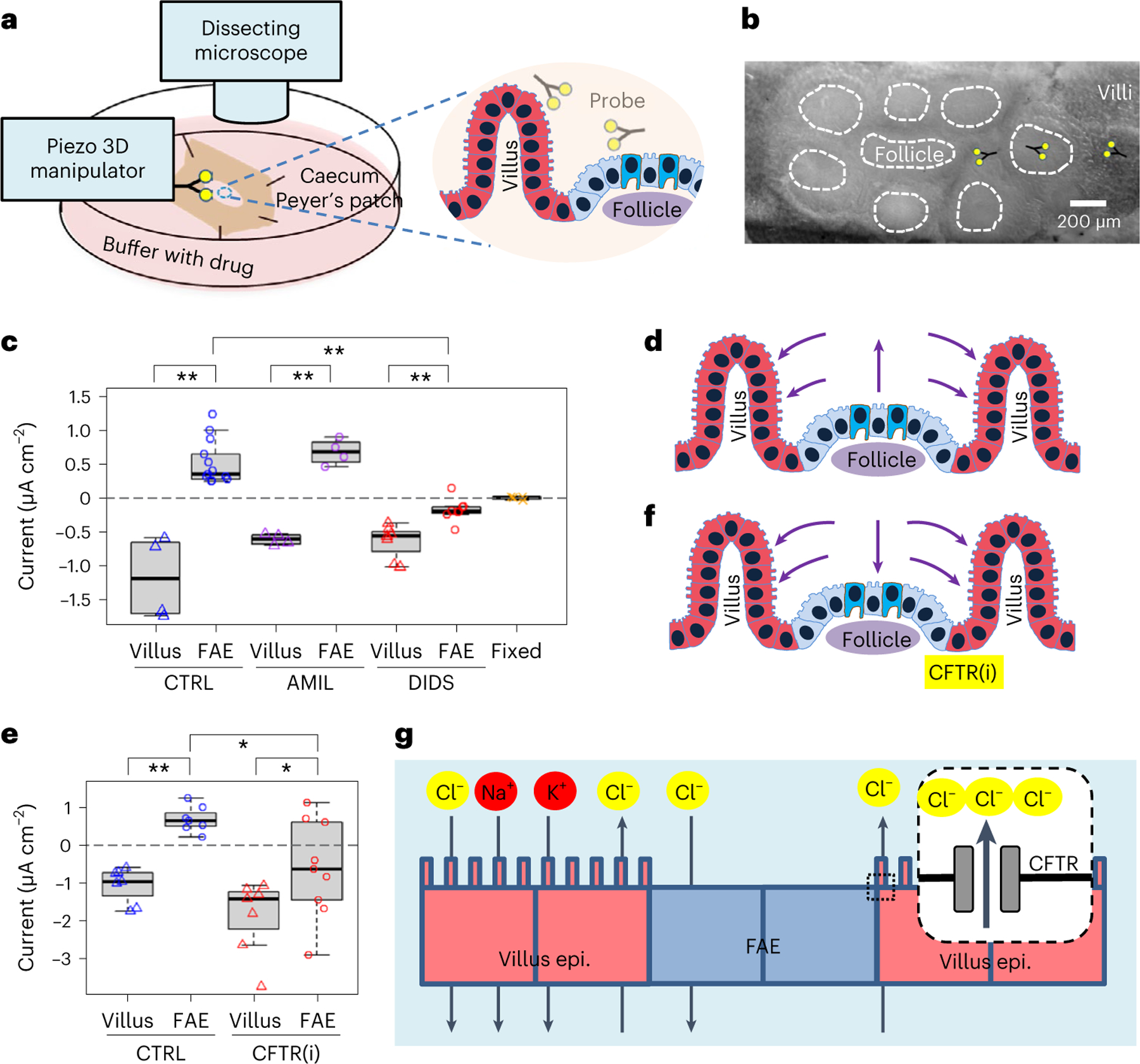
Robust ionic currents emerge from ion channel activities at murine caecum epithelia. **a**, Schematic of the experimental setup. Forks indicate vibrating probes and the sites where current densities were measured. **b**, A mouse caecum under a dissecting microscope as viewed from the luminal side, showing an intact Peyer’s Patch containing a cluster of follicles (dashed enclosures) surrounded by villi. Forks indicate vibrating probes and the sites where current densities were measured. **c**, Peak ionic current densities (*J*_*I*_) in the absence (CTRL) or presence of a general ENaC inhibitor (AMIL) or chloride channel inhibitor (DIDS). Formalin-fixed mouse caeca (‘Fixed’) served as control. Each data point represents the average of 3 to 5 FAE or villus epithelium from each mouse (*n* = 4, 13, 4, 4, 7, 8, 4, respectively, from left to right). **P < 0.01 by one-way ANOVA with post hoc Tukey HSD test. Box tops indicate the 75th percentile, box bottoms indicate the 25th percentile, centre lines indicate median, and whiskers indicate maximum and minimum. **d**, A cartoon depicts ionic flows in caecal FAE and around villus epithelium as detected by vibrating probes. Arrows indicate the flow directions and sizes are approximate. **e**, Peak ionic current density (*J*_*I*_) in the absence (CTRL) or presence of a CFTR inhibitor (CFTR(i)). Each dot represents the average of 3 to 5 FAE or villus epithelium from each mouse (*n* = 7, 7, 7, 9, respectively, from left to right). Box tops indicate the 75th percentile, box bottoms indicate the 25th percentile, centre lines indicate median, and whiskers indicate maximum and minimum. *P < 0.05, **P < 0.01 by one-way ANOVA with post hoc Tukey HSD test. **f**, Ionic flows in the presence of a CFTR inhibitor (CFTR(i)). Note the reversed ionic flow around the FAE due to reduced secretion of chloride or bicarbonate. **g**, Schematic illustrating critical roles of major ion channels and CFTR in generating the ionic flows around the FAE.

**Fig. 3 |:**
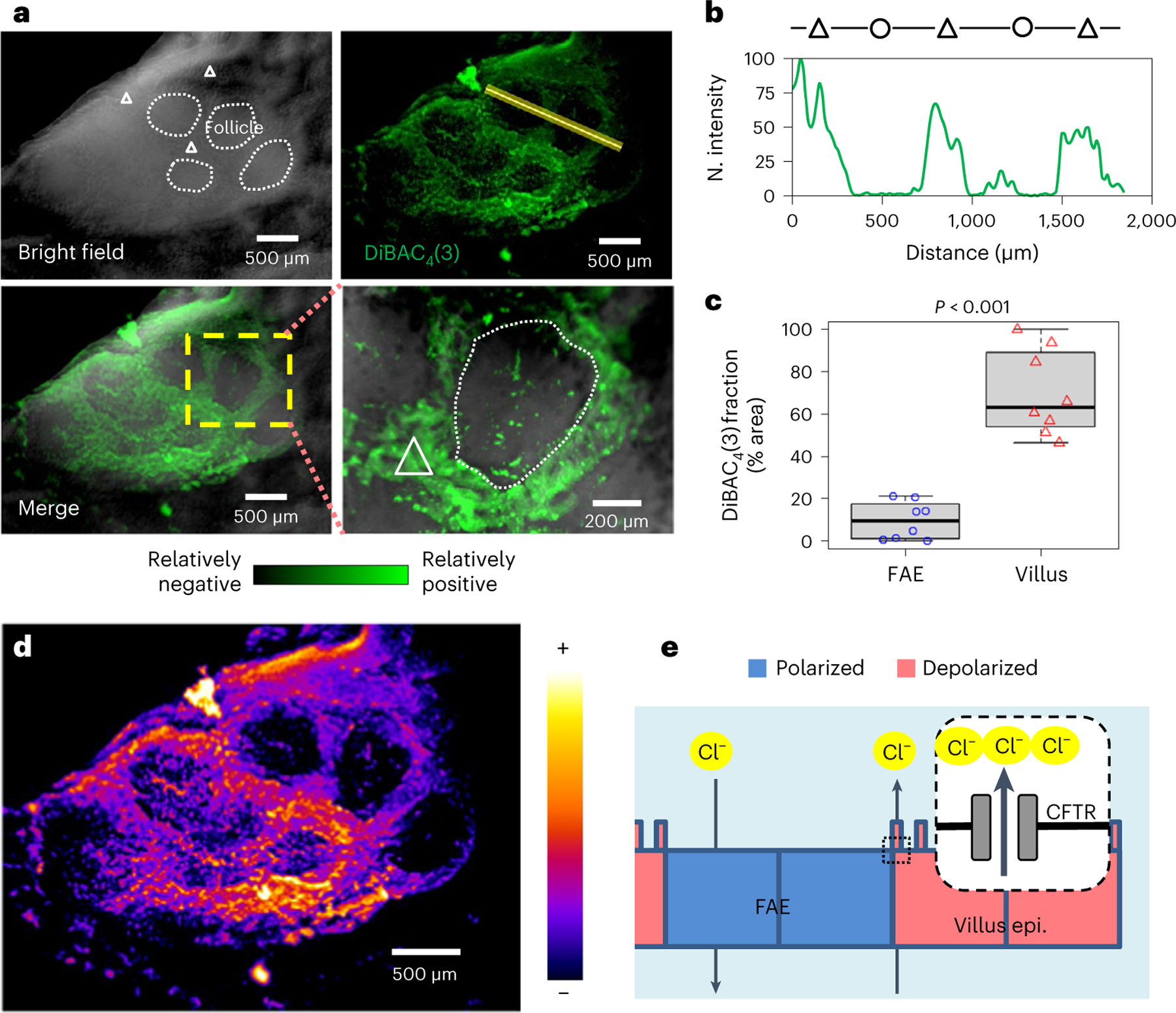
Regional pattern of cell membrane potentials in the FAE and villus epithelium. **a**, Bright-field, live fluorescence and merged images of a mouse caecum, showing a Peyer’s patch stained with membrane potential-sensitive probe DiBAC_4_(3) (also see Extended Data Fig. 3). Enlargement of the yellow dashed area (bottom right panel) highlights a follicle (white dotted enclosure) surrounded by densely stained villus epithelium (white triangle), showing that the villus epithelium is electrically more positive than the FAE. **b**, Fluorescence intensity profile of the line scan in **a** (top right panel), showing a spatial difference in cellular membrane potential between FAE (circle) and inter- and extrafollicular villus epithelium (triangle). **c**, Relative quantitation of resting *V*_m_ of FAE and villus epithelium by DiBAC_4_(3) fluorescence fraction (%). A higher fraction means a more depolarized area. Quantification was based on observations from multiple FAEs and corresponding villus regions across two independent experiments (*n* = 4 mice, *P* < 0.001 by unpaired, two-tailed Student’s *t*-test). Box tops indicate the 75th percentile, box bottoms indicate the 25th percentile, centre lines indicate median, and whiskers indicate maximum and minimum. **d**, Pseudocoloured map of the Peyer’s patch region in **a** with a fire look-up table scale, showing the electrically negative FAEs surrounded by the relatively more positive villus epithelium. **e**, A cartoon to suggest how CFTR and flow of Cl^−^ or HCO ^−^ influence cellular membrane potential at FAE and surrounding villus epithelium (details in the main text).

Recently, we observed a difference in TEPs between the FAE and surrounding villus epithelium^25^. This led us to hypothesize that a regional electrical field might influence the preferential targeting of pathogens, as *S*. Typhimurium and non-pathogenic *E. coli* migrate differently in response to an electrical field^27,28^. To test this, we mapped the bioelectric activities in murine caecal epithelia (Fig. 2a,b). With a vibrating probe to profile the extracellular current densities (*J*_*I*_)^30^, we recorded net outward currents in the FAE and net inward currents in the surrounding villi (Extended Data Fig. 2a–c). The extracellular currents were 0.527 ± 0.091 μA cm^−2^ (mean ± s.e.) and −0.606 ± 0.040 μA cm^−2^ in the FAE and villi, respectively (*P* < 0.01) (Fig. 2c). These recordings reproduced the current circuit between these functionally different epithelia that we observed in our previous study^25^ (Extended Data Fig. 2d). Next, to dissect the main ionic sources of the current, we perturbed the fluxes of sodium (Na^+^) and chloride (Cl^−^), two essential ions for membrane and epithelial bioelectricity^20,31^. We started by using broad-spectrum Na^+^ and Cl^−^ channel blockers, amiloride^32^ and 4,4’-diisothiocyano-2,2’-stilbenedisulfonic Acid (DIDS)^33^, respectively. While the differences between the FAE and villi were still significant (*P* < 0.01, for both drugs), the current density at the villi was not significantly altered by either drug (*P* > 0.05, compared with the no drug control in both cases). In the FAE, the typical outward current remained in the presence of 10 μM amiloride in mouse Ringer’s solution, but it was significantly decreased, reversing to an inward current of −0.173 ± 0.060 μA cm^−2^ (mean ± s.e.) when bathed with 200 μM DIDS in mouse Ringer’s solution (*P* < 0.01, compared with the no drug control) (Fig. 2c). On the basis of these measurements, we conclude that: (1) regional ionic currents loop by entering the absorptive villi and exiting the FAE (Extended Data Fig. 2e); (2) the sustained ionic currents depend on active channel function prevailing in the mucosal epithelium because ionic currents were absent in fixed tissues (Fig. 2c); (3) the large inward current reflects the collective absorption of major electrolytes (Na^+^, K^+^, Cl^−^ and so on) in the villus epithelium and the outward current in FAE results from chloride conductance (Fig. 2d). Thus, we further explored the chloride dependency in gut bioelectricity.

### Regional ionic current flow is CFTR regulated

We sought to determine which Cl^−^ channel has a major contribution to the circuit. CFTR acts as an anion channel^22,34^ that is involved in the osmotic balance of the mucus via the efflux of Cl^−^ anions from the epithelia in many systems, including the intestines^35^. In the intestine, CFTR mediates Cl^−^, HCO ^−^ (bicarbonate) and fluid secretion, with bicarbonate neutralizing luminal acidity. We hypothesized that differential activity of the CFTR could underlie the current loop in the caecum epithelia. We blocked this channel with a selective CFTR inhibitor (10 μM CFTR(inh)-172 (ref. 36) in mouse Ringer’s solution) and measured *J*_*I*_ in both epithelia. While the villi maintained a more widely ranged (−1.066 to −3.740 μA cm^−2^) and robust inward current (−1.875 ± 0.371 μA cm^−2^, mean ± s.e.), the FAE reversed its current from outward to inward (−0.602 ± 0.432 μA cm^−2^) (*P* < 0.05, compared with the no drug control) (Fig. 2e). This pattern is similar to that observed with the use of the generic Cl^−^ channel blocker DIDS (which also inhibits CFTR^37^) (Fig. 2c), showing that CFTR is a key contributor to the Cl^−^ flux and, consequently, to the overall electric current circuit. Taken together, this means that a de facto current circuit is dependent on, or at least regulated by the CFTR-driven Cl^−^ efflux (Fig. 2f). Although the hierarchical approach (first broad-spectrum and then specific Cl^−^ channel inhibitors) points to a role of Cl^−^ flux, we cannot exclude the transport of HCO ^−^ by CFTR as an additional contributor, to some extent, to the currents detected (Fig. 2g). The reversal of the currents in FAE is also supported by the CFTR expression profile since CFTR expression is increased in mucosal epithelial cells that are near lymph nodules^35^.

### Spatial *V*_m_ patterns mirror opposing ionic flows

**Extended Data Fig. 3 |:**
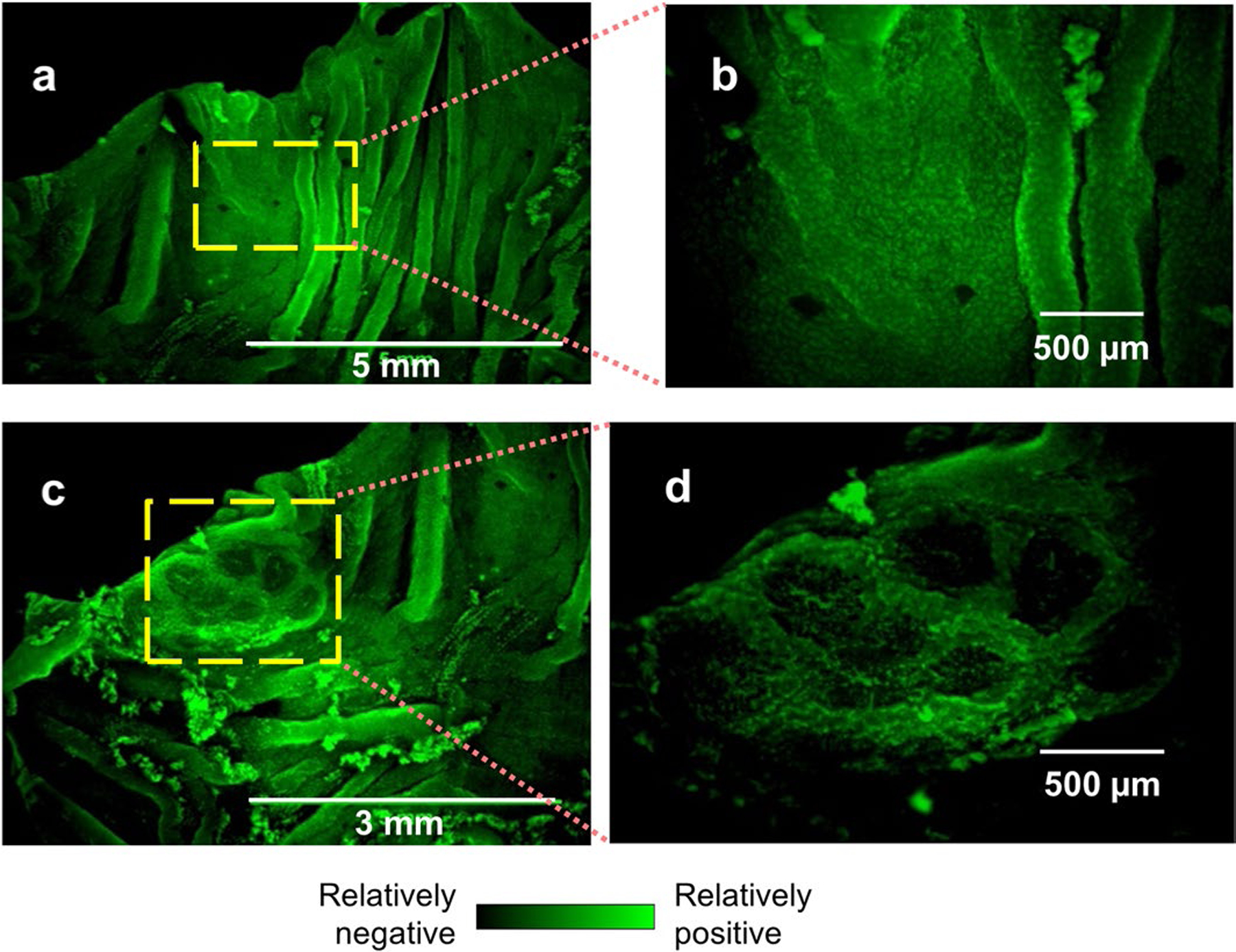
Regional *V*_*m*_ patterns of mouse caecum epithelia. **a**, A low-resolution image of villi away from Peyer’s patch, stained with membrane potential-sensitive probe DiBAC_4_(3). Bar, 5 mm. **b**, A close-up of the checked area in (**a**). Bar, 500 μm. **c**, A low-resolution image of a mouse caecal Peyer’s patch, stained with membrane potential-sensitive probe DiBAC_4_(3). Bar, 3 mm. **d**, A close-up of the checked area in (**c**) (also shown as in Fig. 3a). Bar, 500 μm.

The ionic currents at the tissue level suggest that the enteric cells constituting different epithelia per se could have a spatially different membrane potential. Current can traverse epithelia via paracellular (between cells) and/or cellular (traversing cells) paths^20^. If electrogenic ion flux flows through a cell, variations in *V*_m_ will occur. To test whether FAE and villi have differential *V*_m_, we used the voltage-sensitive dye DiBAC_4_(3)^38^. After incubation with the dye, we imaged a homogeneous polarization in the mouse caecum (Extended Data Fig. 3a,b), except for the Peyer’s patch (Extended Data Fig. 3c,d). Specifically, within the Peyer’s patch, we observed relatively positive (that is, depolarized) potentials in the villi, and relatively negative (that is, polarized) potentials in the FAEs (Fig. 3a). These differences are reliable within the same and across different mouse Peyer’s patches (*P* < 0.001) (Fig. 3b,c). Interestingly, the live dye evidenced well-defined intercellular zones of similar relative potential, negative at the FAEs relative to the positive villi (Fig. 3d). This indicates that while traversing the tissue during their circuit, ions move through the cells (rather than in an exclusively paracellular pathway), which alters their membrane potential. Importantly, the *V*_m_ profile matches the anionic efflux from the CFTR at the villi (Fig. 2e–g). A steady efflux of negative charges from the villi renders a more depolarized *V*_m_ and a steady influx of negative charges into the FAEs will maintain a more polarized *V*_m_ (Fig. 3e). Therefore, a regional *V*_m_ pattern (Fig. 3e) mirrors the ionic currents (Fig. 2g) as the electrogenic anionic charges flow through the cells.

### Lateral bioelectric fields between FAE and villus epithelium

**Extended Data Fig. 4 |:**
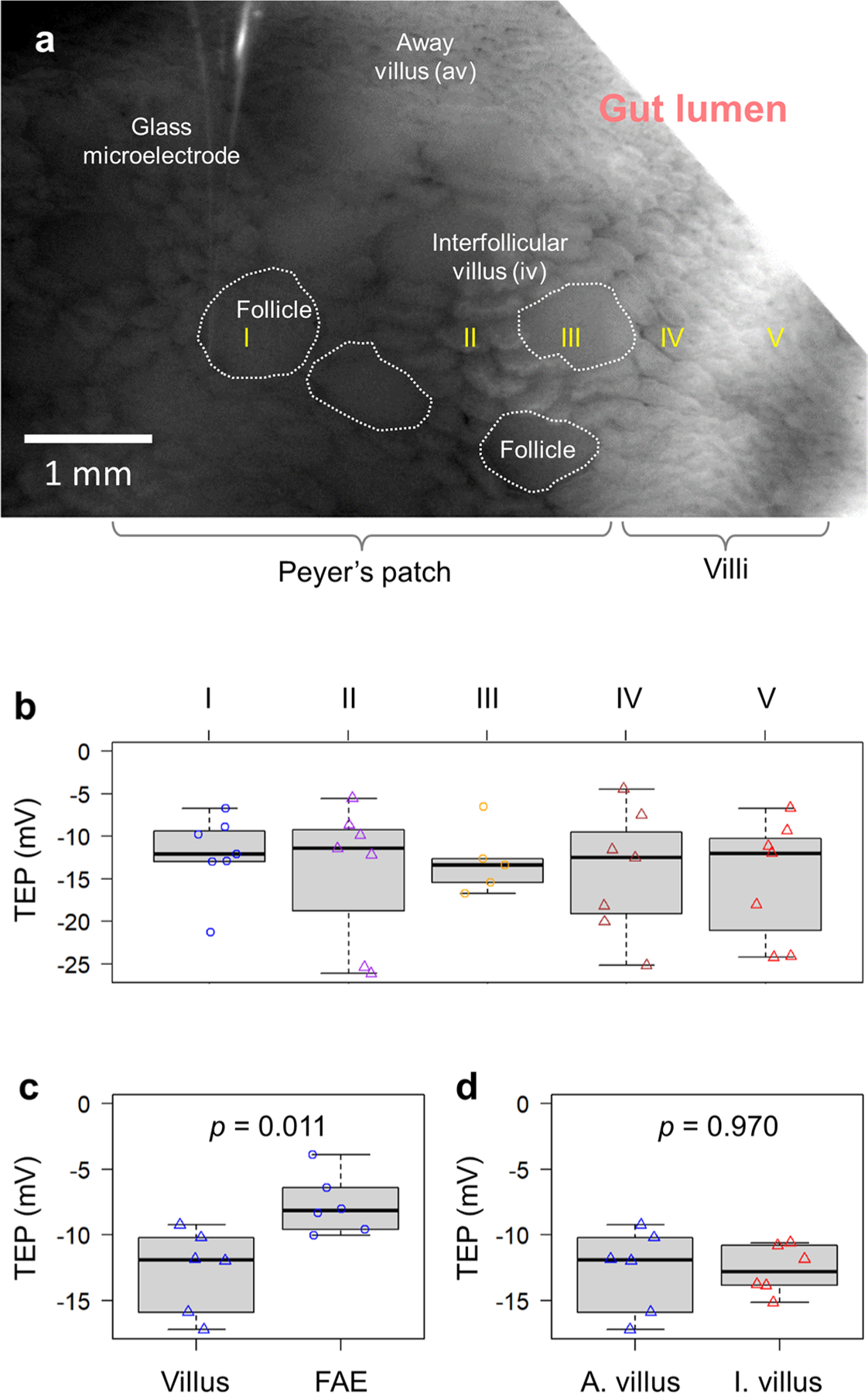
Map of spatial TEP around rat ileal Peyer’s patches and surrounding villi. **a**, A rat ileum under a dissecting microscope, showing a glass electrode approaching a follicle (white dashed enclosure) inside a Peyer’s patch. Bar, 1 mm. **b**, All TEP were negative to the lumen and spatially different as defined by the locations marked in (**a**). Each data point represents a single measurement of a representative rat sample (n = 7, 7, 5, 7, 7 respectively, from left to right). This panel is for demonstration and is not subject to a statistical analysis. **c**, TEPs in the villi are significantly larger than those in FAE (*p* = 0.011 by unpaired, two-tailed Student’s *t*-test). Each data point represents an average of 3 to 5 measurements of each rat sample (n = 6). **d**, The major villus away from FAE indicated as “av” in (**a**) and the interfollicular villus surrounding FAE indicated as “iv” in (**a**) have similar TEPs (*p* = 0.970 by unpaired, two-tailed Student’s *t*-test). Each data point represents an average of 3 to 5 measurements of each rat sample (n = 6). For panels (**b**), (**c**) and (**d**) box tops indicate the 75th percentile, box bottoms indicate the 25th percentile, centre lines indicate median, and whiskers indicate maximum and minimum.

**Fig. 4 |:**
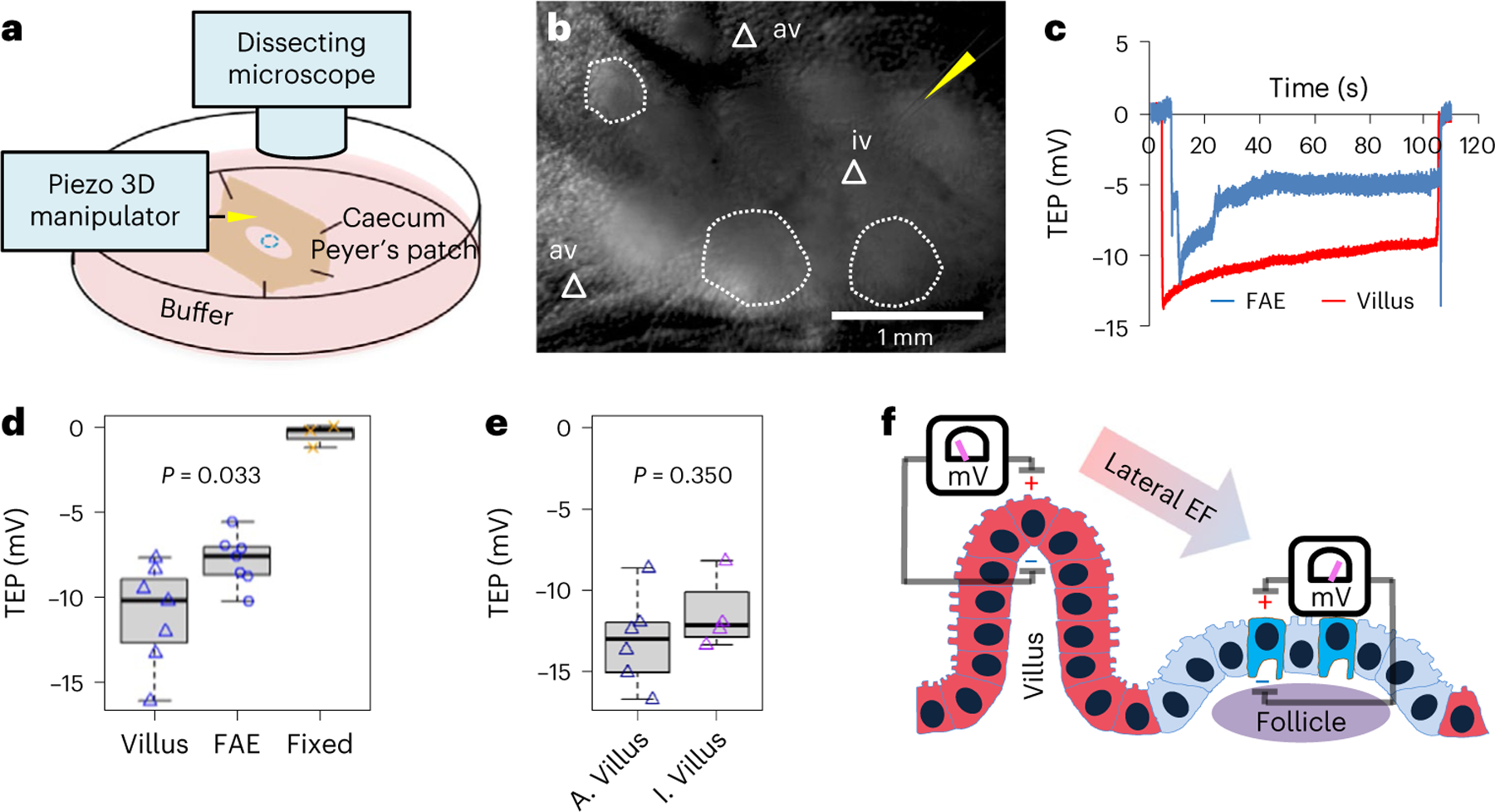
Spatial difference in transepithelial potential generates a lateral potential gradient between FAE and villus epithelium. **a**, A cartoon depicting the TEP experiment setup. **b**, A mouse caecum under a dissecting microscope, showing a glass electrode (yellow arrowhead) approaching an interfollicular villus (white triangle) surrounding a follicle (white dotted enclosure). **c**, Typical TEP traces recorded in the FAE or villus epithelium. **d**, The basal TEP of both villi and FAE were negative in the mouse caeca and significantly larger in the villi than in FAE (*P* = 0.033, by unpaired, two-tailed Student’s *t*-test). Each data point represents the average of 3 to 5 FAE or villus epithelium from each mouse (*n* = 7). Formalin-fixed mouse caeca (‘Fixed’) served as control (*n* = 3), which is not subjected to statistical analysis. **e**, The major villus away from FAE indicated as ‘av’ in **b** (*n* = 6) and the interfollicular villus surrounding FAE indicated as ‘iv’ in **b** (*n* = 4) have similar TEPs (*P* = 0.350, by unpaired, two-tailed Student’s *t*-test). For **d** and **e**, box tops indicate the 75th percentile, box bottoms indicate the 25th percentile, centre lines indicate median, and whiskers indicate maximum and minimum. **f**, Schematic illustration of the spatially distinctive TEPs and the generation of a lateral bioelectric field between FAE and surrounding villus epithelium.

The observed pattern of extracellular electric currents suggested a regional lateral electrical field with the cathode in the FAEs and the anode in the neighbouring villi (Extended Data Fig. 2e). To complete the overall circuit, these extracellular currents must be balanced in subepithelial current corridors that, from Ohm’s law^20^, can only emerge in the presence of voltage drops underneath the FAEs and villi. To test this, we measured TEP by positioning glass microelectrodes^39^ in the FAE and villi of an ex vivo mouse caecum model (Fig. 4a,b). We recorded a significant gradient of inside-negative TEP in FAEs and the surrounding villi, with a larger potential in the latter (*P* = 0.033) (Fig. 4c,d). Similar recordings in rat ileal epithelium and Peyer’s patch reproduced this differential TEPs in the FAE and villi (*P* = 0.011) (Extended Data Fig. 4a–d). The polarity of the TEP is relative to the reference microelectrode, located in the bathing media; with this, we measured an inside-negative TEP. Crucially, there is a consistently larger potential in the villi than in the FAEs, demonstrating a lateral voltage drop that fuels the luminal and subepithelial currents (Fig. 4d and Extended Data Fig. 4c). As for *J*_*I*_, TEP is an active bioelectrical property of epithelia because they are abolished in fixed tissues (Fig. 4d). We also profiled the TEP across the Peyer’s patch and found that the interfollicular villi and villi away from the follicles have a similar TEP in both mouse (*P* = 0.350) (Fig. 4e) and rat (*P* = 0.970) (Extended Data Fig. 4d). Taken together, our extracellular and transepithelial data suggest that the subepithelial current flows from the villi towards the FAE, then exits FAE and enters the villi (Fig. 2d), completing the circuit and generating a local lateral electrical field in the gut mucosa (Fig. 4f).

### *E. coli* and *S*. Typhimurium galvanotax in opposing directions

**Fig. 5 |:**
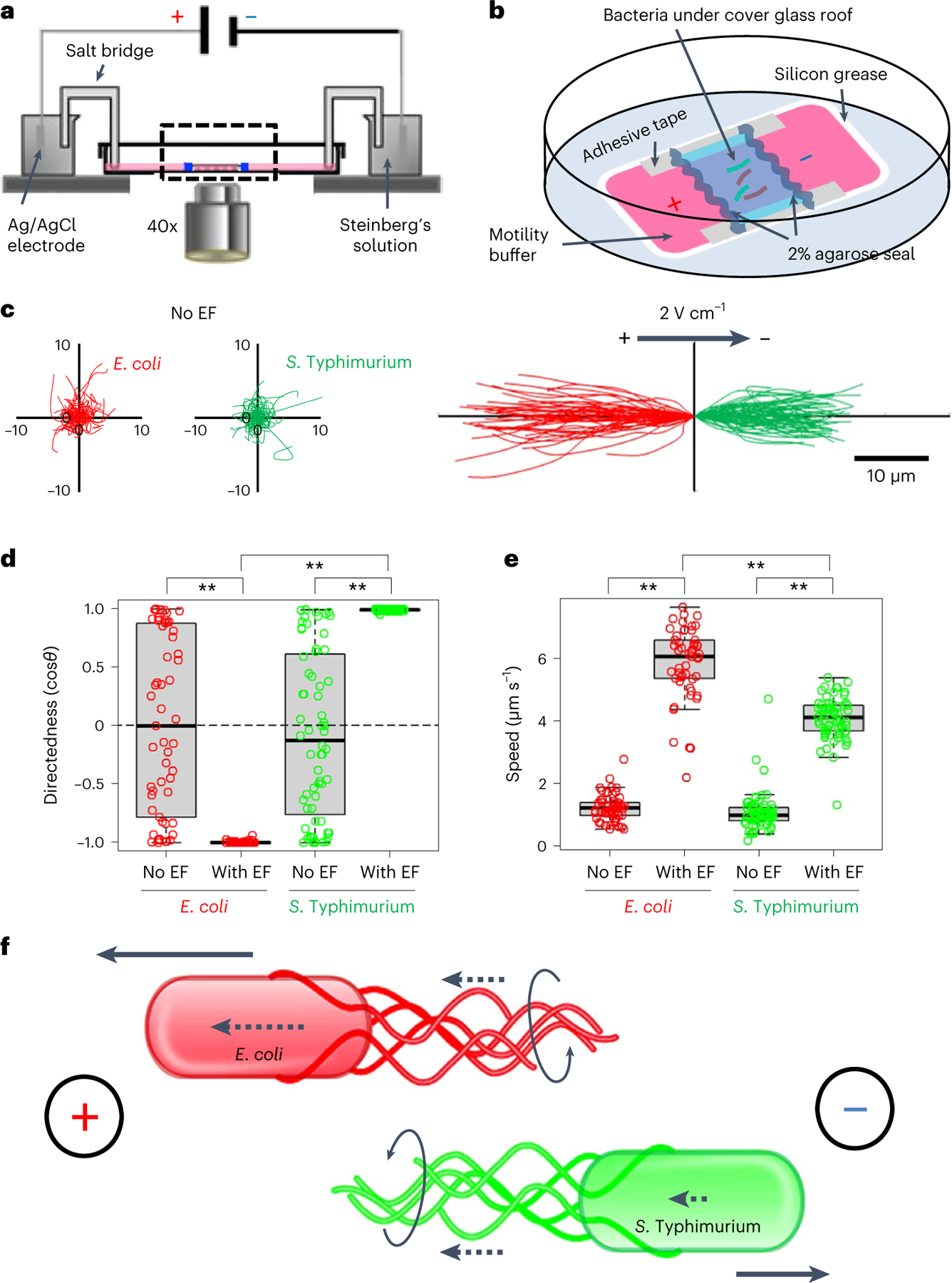
A physiological electrical field drives opposing directional migration of *S*. Typhimurium to the cathode and *E. coli* to the anode in vitro. **a**, Experimental setup. **b**, Enlargement of the dashed area in **a**. **c**, Migration trajectories over 6 s of *E. coli* (red) and *S*. Typhimurium (green) in the absence (No EF) or presence of an electrical field (2 V cm^−1^) with the field polarity as shown. **d**,**e**, Quantification of directedness (cos*θ*: negative to the anode or left, positive to the cathode or right) (**d**), and migration speed (μm s^−1^) (**e**) of *E. coli* and *S*. Typhimurium in the absence (No EF) or presence (With EF) of electrical field. Each circle represents an individual cell (*n* = 57, 53, 64, 65, respectively, from left to right). Box tops indicate the 75th percentile, box bottoms indicate the 25th percentile, centre lines indicate median, and whiskers indicate maximum and minimum. **P < 0.01, by multiple unpaired, two-tailed Student’s *t*-test. **f**, The bacterial surface’s electrical property and flagellar propelling action determine migration direction in the galvanotaxis of *E. coli* and *S*. Typhimurium. Model based on ref. 28 and this work. Dotted arrows indicate the direction and relative size of passive electrophoretic motilities of either bacterial bodies or flagellar filaments. Solid arrows indicate the direction and relative speed of bacterial migration under a 2 V cm^−1^ electrical field in the shown polarity. Circular arrows indicate flagellar rotations in the counterclockwise direction propelling the bacteria along a straight trajectory.

The presence of a regional electrical field raises the interesting possibility of galvanotaxis-driven targeting of local enterobacteria. To test this, we first selected well-established representatives of commensal and pathogenic bacteria, *Escherichia coli* and *S*. Typhimurium, respectively^25,40^. Next, we subjected these bacteria to an endogenous-like electrical field in vitro (Fig. 5a,b), either sequentially (Supplementary Videos 1 and 2) or simultaneously (Supplementary Video 3). Without an electrical field, both types of bacteria migrate randomly (Fig. 5c), with their averaged directedness (<cos*θ*>, defined in Methods) values close to 0 (Fig. 5d). In the presence of an electrical field, *E. coli* cells presented a directedness of −0.995 ± 0.001 (mean ± s.e.) and *S*. Typhimurium a directedness of 0.994 ± 0.001 (Fig. 5d), showing a robustly biased migration of all cells towards the anode and cathode, respectively (Fig. 5c). The migratory speed (spanned distance over elapsed time) of both bacteria was around threefold faster in the presence of an electrical field (*P* < 0.01, *E. coli* or *S*. Typhimurium with electrical field compared with no electrical field). Intriguingly, *E. coli* migrated significantly faster than *S*. Typhimurium (5.848 ± 0.158 μm s^−1^ versus 4.083 ± 0.083 μm s^−1^ (mean ± s.e.), respectively) in the presence of an electrical field (*P* < 0.01) (Fig. 5e). Therefore, the O-antigen-deficient *E. coli* K12 and the smooth, virulent *S*. Typhimurium 14028S have opposing responses to the same electric cue (Supplementary Video 3).

Motile bacteria swim directionally by arranging their filament flagella on one end of the cell (the prospective back) in a bundle. These flagella rotate counterclockwise to propel the bacteria along a straight trajectory^41^ by generating a thrust force in the piconewton range^42^. To further explore the role of flagella in the galvanotaxis of *S*. Typhimurium, we tested a flagellar mutant strain (*∆fliC*, *fljB*::MudJ)^43^. Most of these mutants were non-motile and unresponsive to the applied electrical field, confirming that flagella are indeed essential for electrical field-guided galvanotaxis in *S*. Typhimurium^27^ (Supplementary Video 4). To investigate whether an applied electrical field can induce passive electrophoretic movement of the *S*. Typhimurium flagellar bundle ahead of the cell body, we initiated our examination with latex beads. These negatively charged beads exhibited slow migration towards the anode under our galvanotaxis experiment conditions, as observed at specific focal planes (Supplementary Video 5). Subsequent staining of *Salmonella* flagella, pre and post electrical field application, was carried out using antibody targeting *Salmonella* O- and H-antigens (Supplementary Table 1). In the absence of an electrical field, the flagella exhibited random orientation. However, upon electrical field exposure, the flagella predominantly repositioned to the anode side, trailing the bacterial body (Extended Data Fig. 6a–e). These findings not only reinforce Adler’s model^28^ but also elucidate a potential mechanism for directional galvanotaxis in motile *Salmonella* via its flagella (Fig. 5f).

To test whether other commensal bacteria can respond to an applied electrical field in vitro, we conducted galvanotaxis assays with *Bacillus subtilis* (*B. subtilis*). Surprisingly, this commensal did not perform robust galvanotaxis (Extended Data Fig. 7a and Supplementary Video 6), despite being biased towards the cathode (directedness with electrical field vs no electrical field: 0.385 ± 0.081 vs 0.117 ± 0.095 (mean ± s.e.), *P* = 0.034) (Extended Data Fig. 7b). Unlike *S*. Typhimurium that migrated straight towards the cathode (Fig. 5d) with increased speed (Fig. 5e), the migration speed of *B. subtilis* did not increase (electrical field vs no electrical field: 0.567 ± 0.030 vs 0.558 ± 0.028 μm s^−1^ (mean ± s.e.), *P* = 0.839) (Extended Data Fig. 7c) and remained one order of magnitude slower than that of *S*. Typhimurium (Fig. 5e). Hence, *B. subtilis*, one of the most abundant commensals in the human gut, do not undergo robust directional migration when exposed to a small electrical field.

The bacterial galvanotaxis is not, or at least not fully due to electrophoresis since both *E. coli* and *S*. Typhimurium are negatively charged and they migrated to the anode in a higher electrical field when fixed by formaldehyde^28,44^; neither is it due to fluid flow because our experiments were conducted in sealed microfluidic chambers (Fig. 5b). On the basis of these data, we hypothesize that the *E. coli* K12 and the enteric pathogen *S*. Typhimurium 14028S may act and move differentially in the vicinity of the intestinal epithelia in response to an existing, naturally occurring bioelectrical signal.

### *S*. Typhimurium galvanotaxis is independent of chemotaxis

Previous research has shown that *S*. Typhimurium invades the murine ileum Peyer’s patches by detecting gradients of host-derived chemoattractants. This process was contingent upon the flagellar apparatus and specific chemotaxis protein receptors^11,45^. To probe the role of chemotaxis in galvanotaxis-facilitated migration, we executed an in vitro galvanotaxis assay with a chemotaxis-deficient mutant in the background of the *S*. Typhimurium 14028S, specifically lacking the methyl-accepting chemotaxis protein CheB^46^. This mutant displayed marked directional migration towards the cathode (Supplementary Video 7), aligning with the movement pattern of the wild-type strain (Supplementary Video 2). This suggests that CheB is not crucial for bacterial galvanotaxis^27^ and that the mutant might still navigate effectively to the FAE. Subsequently, mouse caecum explants were exposed to a 1:1 mixture of *E. coli* (K12, dTomato-expressing) and an *S*. Typhimurium wild-type strain (14028S, EGFP-expressing). A comparable experiment was set up with another *S*. Typhimurium wild-type strain (mCherry-expressing) against either the *cheB* mutant (EGFP-expressing) or a non-motile flagellar mutant (EGFP-expressing) as controls. At 30 min post incubation, epithelium-associated bacteria were recovered from FAEs isolated using fine biopsy punches. Quantitative analysis revealed that the *S*. Typhimurium recovery rate was about five times that of the *E. coli* from the FAE (*P* = 0.063) (Extended Data Fig. 8a). The flagellar mutant exhibited a 20-fold lower recovery than its wild-type counterpart (*P* = 0.021) (Extended Data Fig. 8a), underscoring the pivotal role of flagella in congregating at the FAE. Importantly, the *cheB* mutant recovery rate mirrored that of the wild-type *S*. Typhimurium (*P* = 0.937) (Extended Data Fig. 8a), suggesting that, unlike flagella, CheB is inconsequential in this bioelectricity-driven event in our ex vivo setup. The competitive index analysis and its subsequent data comparison (*P* = 0.019) (Extended Data Fig. 8b) further substantiate this notion.

### CFTR modulates *S*. Typhimurium localization

**Fig. 6 |:**
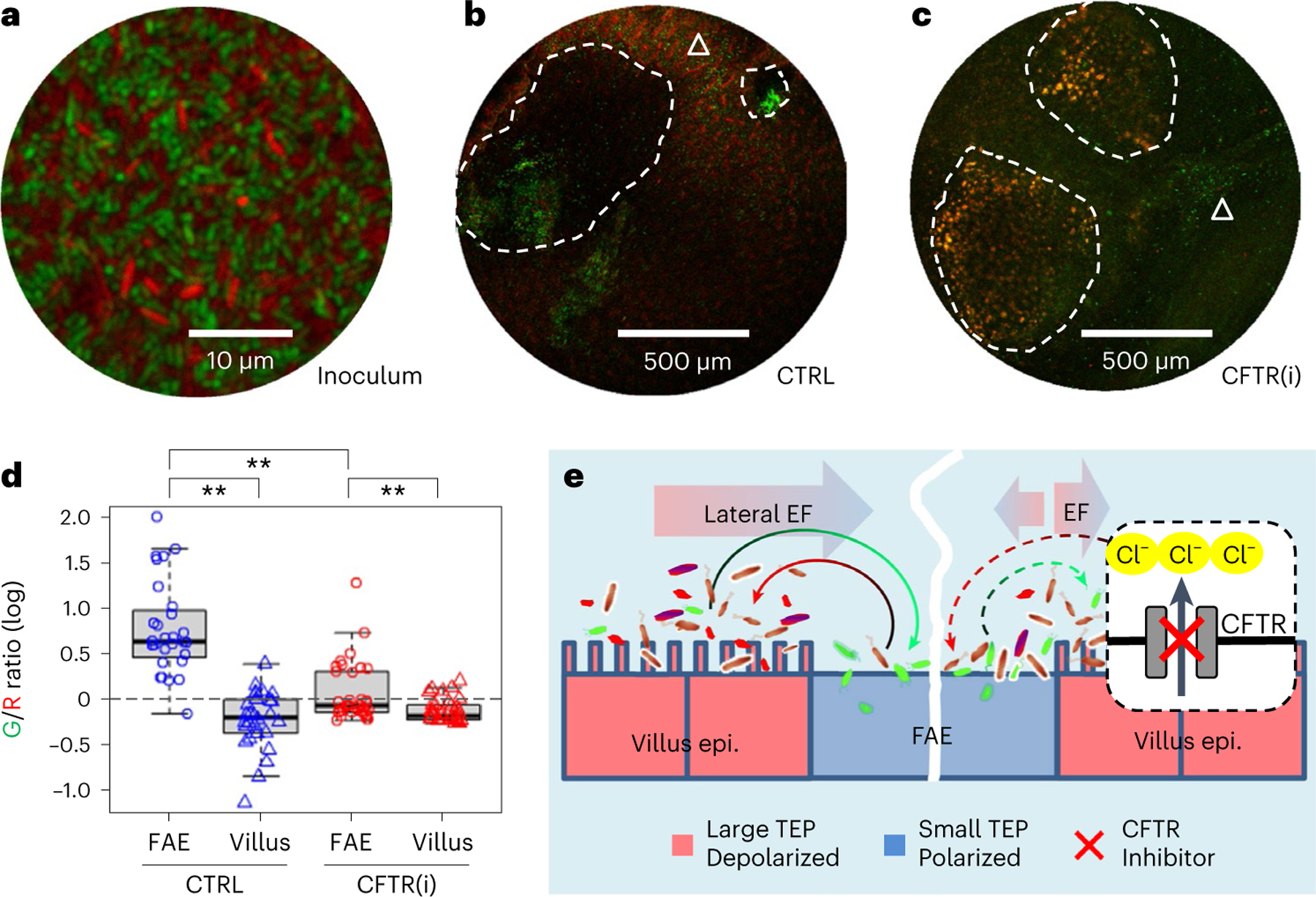
Inhibiting CFTR decreases *S*. Typhimurium recruitment to the FAE and increases *E. coli* recruitment. **a**, A confocal image illustrates the inoculum of *E. coli* K12 (red) and *S*. Typhimurium 14028S (green) at a 1:1 ratio (10^8^ c.f.u.s ml^−1^ in mouse Ringer’s solution). **b**, A representative confocal image shows *S*. Typhimurium (green) accumulating in the FAE (white dashed enclosure) and *E. coli* (red) dominating in the villi (white triangle). **c**, A representative confocal image exhibits *S*. Typhimurium (green) and *E. coli* (red) co-existing in multiple regions of the FAE (white dashed enclosure) and the villus epithelium (white triangle) when CFTR is inhibited (CFTR(i)). **d**, Quantification of mean green/red fluorescence intensity ratios associated with FAE or villus epithelium in common logarithm. Analysis was based on observations from multiple FAEs and corresponding villus regions across two independent experiments (*n* = 4 mice). Box tops indicate the 75th percentile, box bottoms indicate the 25th percentile, centre lines indicate median, and whiskers indicate maximum and minimum. Dashed line indicates the ratio of 1. **P < 0.01, by one-way ANOVA followed by post hoc Tukey HSD test. **e**, A model proposing that a local bioelectric network drives *S*. Typhimurium targeting to the FAE entry port through galvanotaxis in gut epithelia. A lateral electrical field emerges from spatially defined bioelectric activities (ionic flow, cellular membrane potential and TEP), allowing the establishment of a microbioelectric route between the anatomically and functionally different FAE and villus epithelium. This not only favours the *S*. Typhimurium (green) to navigate to the FAE (curved green arrow) but also prevents the *E. coli* (red) from accidentally entering this ‘danger zone’ (curved red arrow). Inhibiting CFTR blocks Cl^−^ efflux (red cross) in the enteric epithelium near FAE, reducing or reversing ion flow in the FAE, resulting in increased *E. coli* recruitment (curved red dashed arrow) to the FAE or directing the *S*. Typhimurium (curved green dashed arrow) towards the adjacent villus epithelium.

Having revealed the CFTR-regulated regional electrical fields, demonstrated the opposing directional migration of *E. coli* and *S*. Typhimurium under physiological electrical fields, and established that *S*. Typhimurium galvanotaxis operates independently of chemotaxis, we next investigated whether disrupting the endogenous electrical fields would affect *S*. Typhimurium localization in the FAE. To test this, we performed a competitive tropism assay in our ex vivo mouse caecum model using a mixture of differentially tagged *E. coli* K12 and *S*. Typhimurium 14028S (Fig. 6a). In unperturbed endogenous electrical fields, confocal microscopy revealed that dTomato-tagged *E. coli* is predominantly localized in the villi, avoiding the FAEs, while EGFP-tagged *S*. Typhimurium showed a preference for the cathodic FAEs (Fig. 6b). Quantification of the spatial distribution of *S*. Typhimurium and *E. coli* via a green/red fluorescence intensity ratio confirmed their respective preferences (*P* < 0.01, Fig. 6d). Notably, the bacterial tropism towards anodic villi and cathodic FAEs aligns with the lateral potential gradient (Fig. 4f), the *V*_m_ pattern (Fig. 3d) and the robust directional galvanotaxis in vitro (Fig. 5c). We validated that the bioelectricity-modulated bacterial targeting is an active biological process, by including fluorescently labelled latex beads in the inoculum in a 1:2 (bead/bacteria) ratio (Extended Data Fig. 9a). The beads showed a relatively homogeneous distribution in the FAEs and villi (Extended Data Fig. 9b,c) and, when subjected to fluorescence intensity and ratiometric analysis, revealed the asymmetric bacterial distribution (Extended Data Fig. 9d,e). Finally, upon electrical field perturbation with CFTR inhibitor, we observed a significant decrease in the *S*. Typhimurium vs *E. coli* fluorescence intensity ratio (*P* < 0.01 compared with the no drug control, Fig. 6c,d) in the FAE. This could indicate either a reduction of *S*. Typhimurium recruitment to the FAE or an increase in *E. coli* recruitment, or both. Our data support the latter scenario since there was elevated dTomato-tagged *E. coli* signal in the FAE and elevated EGFP-tagged *S*. Typhimurium targeting to the adjacent villus epithelium (that is, the villus surrounding an FAE, Fig. 6c), decreasing the amount available to amass in the FAE. The CFTR inhibition did not alter the distribution on the absorptive villi far from the FAE (*P* = 0.066 compared with the no drug control, Extended Data Fig. 10a–c).

Taken together, these findings suggest that *S*. Typhimurium recruitment to the FAE, as demonstrated in our ex vivo model, does not occur through bacterial chemotaxis receptor-engaged pathways, but rather via a CFTR-regulated bioelectrical configuration that drives the differential taxis of both *S*. Typhimurium and *E. coli* targeting the gut epithelium (Fig. 6e).

## Discussion

How pathogenic bacteria reach a vulnerable yet elusive entry port to challenge the host immunity is a fundamental issue in bacterial pathogenesis. Using energy taxis, motile bacteria can invade the ileal Peyer’s patch^11,45,47^, a behavioural trait that is mediated by the flagellar motility apparatus and chemotaxis signal transduction pathway^48^. Here we have demonstrated that *S*. Typhimurium navigates to the FAE through a local bioelectric network. This work stems from our original finding that a microbioelectric potential gradient exists between anatomically and functionally different villus epithelium and FAE^25^. We show that in the villi, the robust channel-engaged absorptive function results in an inward current sustained from the uptake of electrolytes. In the FAE, the ionic flow is particularly restricted to Cl^−^ influx, resulting in an outward current (by convention, the current direction is defined by the flow of positive charges) that could be influenced or reversed by the CFTR-driven efflux of Cl^−^ (Fig. 2d,f,g). Spatial distribution and segregation of these electrogenic carriers generates spatially differentiated potentials across membranes (Fig. 3d) and epithelia (Fig. 4f). These allow the establishment of a lateral bioelectric route between the absorption-purposed villus epithelium and the surveillance-purposed FAE, which guides this pathogenic *S*. Typhimurium to the FAE through a galvanotaxis-based mechanism (Fig. 6e).

The bioelectricity-modulated bacterial targeting in the gut epithelium demonstrated in this study is distinct from chemotaxis, as well-characterized chemoreceptor mutants^46^ exhibit directional galvanotaxis (Supplementary Video 7), despite a significant number of these mutants being still present in the Peyer’s patches during the early stage of *S*. Typhimurium infections^11^. While chemotaxis involves bacteria sensing chemical gradients and moving towards or away from specific compounds, directional migration in response to a voltage gradient either to the cathode or anode primarily depends on the bacteria’s saccharide compositions and surface electric properties^28,49^. However, both directional cues could coexist for several reasons: (1) both chemotaxis and galvanotaxis in bacteria rely on flagellar motility machinery; (2) an existing electrical field could contribute to the establishment of chemoattractant gradients; (3) likewise, spatial distribution and segregation of electrogenic chemoattractants or chemorepellents could contribute to the formation of potential gradients. As a result, our model (Fig. 6e) is not mutually exclusive with respect to energy taxis^11,45^, but instead suggests an alternative and/or complementary mechanism in modulating *S*. Typhimurium targeting to the gut epithelium.

Given the prevalence of mucosal-dwelling pathogens, other enteropathogenic bacteria might also utilize the local bioelectrical blueprint as a navigational strategy and engage in galvanotaxis in vivo. Notably, the bioelectric field configuration in the gut epithelia not only favours the *S*. Typhimurium to be sorted to the FAE but also prevents *E. coli* from accidentally entering it (staying away from the ‘danger zone’) (Fig. 6e). This highlights the biological and clinical relevance of the bioelectric field. If, for genetic, environmental or other reasons, the local bioelectric field is improperly configured (polarity reversal or shift in magnitude), it may trap *E. coli* or other commensals more often and generate hyper-immunity and/or autoimmunity to the gut microbiota. For instance, the root cause of inflammatory bowel disease is thought to be an excessive and abnormal immune response against commensal flora in genetically susceptible individuals^50^. It will be interesting to learn whether those susceptible patients have misconfigured bioelectric networks or aberrant bioelectric activities in gut epithelia.

*Salmonella* preferentially invades the epithelium in the FAE of the small intestine while generally being resisted by the colon epithelium. The precise signals governing the preference and resistance are not fully understood^11,51–53^. Even with streptomycin pretreatment^54^, *S*. Typhimurium infrequently breaches the dense mucus barrier in the distal colon. Conversely, the thinly mucus-covered caecum epithelium is more vulnerable to *S*. Typhimurium invasion^55^. Although the repulsive electrostatic properties of dense mucus seem to protect against *Salmonella* colonization in the mouse colon, our model offers a more straightforward explanation: the bioelectrical configuration of the colon epithelium favours commensal flora over *S*. Typhimurium, probably due to the lack of structures such as Peyer’s patches or follicles (Fig. 6e). While our ex vivo model represents a useful tool to study gut epithelial bioelectricity and *Salmonella* pathogenesis, our study has some limitations: the TEPs that we measured using glass microelectrodes were different from those measured by Ussing chambers, which were usually 1–2 mV, positive inside the epithelium^56^. This inconsistency could be due to the different techniques and referencing used since a previous study using a standard calomel electrode detected a positive TEP of 10.86 mV (relative to the lamina propria) in the rat proximal colon^57^. Intriguingly, that study also reported spatially different TEPs in rat colonic epithelium and found that the TEPs were reversed in chemically induced colitis^57^. While our data exempted some Na^+^ channels and implicated the Cl^−^ conductance and the CFTR as a major electrogenic source around the FAE, other ions and channels cannot be definitively ruled out, particularly to explain the inward currents in the villi. Considering that homozygous mouse with the CFTR knockout, which underlies cystic fibrosis disease, can only survive for several weeks due to intestinal obstruction^58^, we expect a change in bioelectric activities in the CFTR knockout mice.

## Methods

### Animals and surgery

C57BL/6 mice (*Mus musculus*, 6–10-week-old) were purchased from Jackson lab. Wistar rats (*Rattus norvegicus*, 6–10-week-old) were purchased from Charles River. Both male and female mice or rats were used for each experiment unless otherwise specified in the figure legend. The rodents were maintained under a strict 12 h light cycle and given a regular chow diet in a specific pathogen-free facility at the University of California (UC), Davis. All animal experiments were performed following regulatory guidelines and standards set by the Institutional Animal Care and Use Committee of UC Davis under protocols 20144 and 23542. In brief, we dissected mouse caecum or rat ileum following euthanasia and opened it longitudinally along the mesenteric attachment remnant to avoid incision damage to a Peyer’s patch. After thorough washing in mouse Ringer’s solution (154 mM NaCl, 5.6 mM KCl, 1 mM MgCl_2_, 2.2 mM CaCl_2_, 10 mM glucose and 20 mM HEPES, pH 7.4) to remove the luminal contents, we placed the specimen with mucous side facing up, on a 30° slope of silicone gel, prepared from polydimethylsiloxane (PDMS) in custom-made measuring chambers. The intestine was aligned and immobilized with fine metal pins before taking measurements. This process was usually completed within 5 min at room temperature^25^.

### Measuring ionic currents with vibrating probes

We used non-invasive vibrating probes to measure the extracellular electric current density (*J*_*I*_, in μA cm^−2^) of mouse caecum epithelium as previously described^30,59^. The probes, platinum-electroplated at the tip (~30 μm ball diameter), vibrated at a frequency between 100–200 Hz. Before measurements, the probe was calibrated to the experimental conditions by an applied *J*_*I*_ of 1.5 μA cm^−2^. Under a dissecting microscope, mounted mouse caeca were positioned in the non-conductive measuring chamber. The plane of probe vibration was perpendicular to the epithelial surface at a distance as close as possible. *J*_*I*_ was recorded until the plateau peak was reached (<1 min). Reference values were recorded with the probe away from the epithelium surface (>>1 mm). A detailed schematic depicting the equipment setup and measuring procedures, as well as a real picture of the probe over the FAE is illustrated in Fig. S3 of our previous publication^25^. Measurements were taken at room temperature in mouse Ringer’s solution. During calibrations and measurements, a Faraday ‘wall’ (grounded aluminum-wrapped cardboard) covered the microscope. As a control, we measured *J*_*I*_ near the surface of formalin-fixed mucous epithelium. Data were acquired and extracted using WinWCP V4 (Strathclyde Electrophysiology Software) and analysed using Microsoft Excel. Box graphs were generated using a standard R script.

### Pharmacological inhibition of ion channels

We measured *J*_*I*_ in the presence of 10 μM amiloride^60^ or 200 μM DIDS^61^, in 20 dissected mouse ceca, in either FAE or villus epithelium. We used a CFTR inhibitor CFTR(inh)-172 (10 μM), as described previously^36,59^. All drugs (Supplementary Table 1) were purchased from Sigma. We incubated the drugs in the indicated working concentration in mouse Ringer’s solution for at least 15 min before conducting a measurement, as described previously^59^.

### Mapping cellular *V*_m_ with DiBAC_4_(3)

To spatially assess *V*_m_ of the intestinal epithelium, we utilized the live fluorescent voltage reporter DiBAC_4_(3)^38^ (Supplementary Table 1). Each caecum was mounted on a flat surface of 2% agarose within Petri dishes. The tissue was then incubated with 2 μM DiBAC_4_(3) in darkness for 30 min. Subsequent imaging involved capturing both bright-field and green fluorescence (using the FITC filter) at various time points with a ZEISS SteREO Discovery.V12 microscope. This microscope was equipped with a Retiga R6 camera, featuring a large 16 mm field of view and high resolution (6 million pixels, 4.54 μm each). Image acquisition was performed using Axiovision software (Carl Zeiss), with further processing in ImageJ.

For analysis, FAEs and corresponding (matched in size and adjacent location) villi were outlined as regions of interest (ROIs) in the bright-field images. These ROIs were then applied to the fluorescence images, enabling quantification and comparison of signals within the same visual field. The relative resting *V*_m_ of either FAE or villus epithelium was determined by analysing normalized mean fluorescence intensities (with higher intensity indicating more depolarization) or by calculating the segmented fluorescent fraction (% area) within each ROI. This calculation was facilitated by thresholding the DiBAC_4_(3) fluorescence signal, taking advantage of its bright and consistent intensities. To ensure reliability, all imaging parameters were standardized across experiments.

### Measuring TEP with glass microelectrodes

We used glass microelectrodes to directly measure the TEP of intestinal epithelium as previously described^39^. TEP was recorded by microelectrode impalement through the epithelial layers. Microelectrodes (1–2 μm tip diameter, 3 M NaCl electrolyte) had resistances of ~1–2 MΩ and the potentials were offset to 0 mV before impalement. FAE and adjacent villus epithelium were discriminated under a dissecting microscope (ZEISS SteREO Discovery.V12) within a Faraday cage on an antivibration table. The potential typically returned to the baseline of 0 mV after microelectrode withdrawal. If the reference baseline was >±1 and ≤±5 mV, the value was subtracted from the TEP recorded; if >±5 mV, the trace was rejected. As a control, we measured the TEP of formalin-fixed mucous epithelium. Measurements were performed at room temperature in mouse Ringer’s solution. Data were acquired (saturated sampling at 100 Hz) and extracted using pClamp 10 (Molecular Devices) and analysed using Microsoft Excel. Box graphs were generated using a standard R script.

### Engineering *E. coli* and *S*. Typhimurium expressing different fluorescent proteins

Plasmids and bacterial strains used in this work are listed in Supplementary Table 1. The commensal *E. coli* K12 expressing red fluorescent protein (RFP) was made by transforming the laboratory DH5α with plasmid pdTFT/RalFc. The pdTFT/RalFc was constructed in two steps. First, a fragment of a *dTomato* gene coding for a dimeric red fluorescent protein^62^ was amplified with primers of dTomato-F (5’-ACATATGGTGAGCAAGGGCGAGGAGGTC-3’) and dTomato-R (5’-ACCCGGGATGCATTACTTGTACAGCTCGTCCATGCCGTAC-3’). This fragment was then digested with NdeI/NsiI and cloned into NdeI and PstI sites of pFT/RalFc, a low-copy plasmid based on pBBR1-MCS4 (ref. 63).

The *S*. Typhimurium strain derived from IR715 that constitutively expresses mCherry coded in its genome was described previously^25^. The green fluorescent protein (GFP)-expressing *S*. Typhimurium strain was generated by electroporating pGFT/RalFc^25^ into a smooth, virulent IR715, derived from wild-type isolate ATCC 14028S^64^, or a flagellar double knockout mutant (Δ*flic*/*fljB*)^11,43^, or a chemotaxis-deficient mutant (Δ*cheB*)^46^. All the plasmids used in this study were sequenced. Robust and constitutive fluorescent protein expression in both strains was confirmed and visualized under a fluorescence microscope using RFP and GFP filters.

All bacteria were incubated aerobically at 37 °C in Luria-Bertani (LB) broth (per liter: 10 g tryptone, 5 g yeast extract, 10 g NaCl) or on LB agar plates (1.5% Difco agar) overnight. Antibiotics were used at the following concentrations unless stated otherwise: 30 μg ml^−1^ chloramphenicol, 50 μg ml^−1^ nalidixic acid, 100 μg ml^−1^ ampicillin, 50 μg ml^−1^ kanamycin and 10 μg ml^−1^ tetracycline.

### Bacterial galvanotaxis and time-lapse recording

**Extended Data Fig. 5 |:**
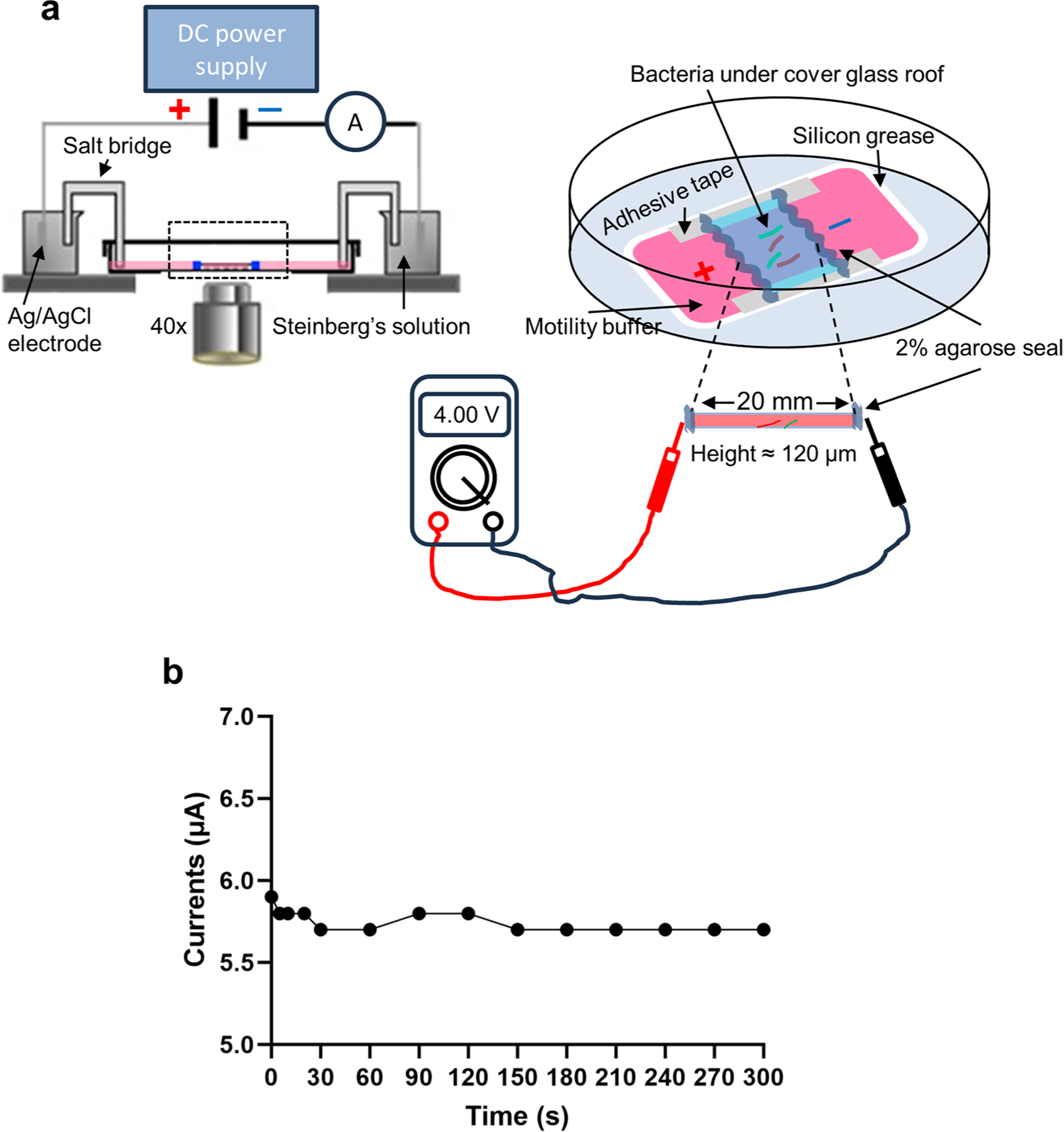
Electric currents remained constant during bacterial galvanotaxis. **a**, A schematic of bacterial galvanotaxis setup. Actual voltage drop is measured across a 20 mm microfluidic chamber that is sealed with 2% agar. Channel height is about 120 μm. **b**, Representative currents in the circuit monitored by an ammeter in series in (**a**) during a five-minute experiment.

Bacterial galvanotaxes were conducted in custom-made glass-bottom chambers using a motility buffer with defined ionic strength and pH^27^. Bacteria or latex beads were capsulated within a microfluidic channel measuring 22 × 20 × 0.12 mm (Fig. 5b and Extended Data Fig. 5a). From our transepithelial potential recordings, a voltage drop of 5–10 mV exists from the villi towards the FAE (Fig. 4d). Considering a distance of 1 to 10 enterocyte diameters (50 μm approximate size per cell), a lateral electrical field of 0.1–2 V cm^−1^ forms from the villi towards the FAE. Then, we empirically selected 2 V cm^−1^ from our initial strength screen and drawing from previous literature^27^ and our accumulated expertise^65^. The strength of 2 V cm^−1^ is equivalent to 4 V across the 2 cm electrotactic channel (or 200 mV mm^−1^) and was used for most of our experiments, unless otherwise stated. Before initiating each experiment, the actual voltage drop was verified using a voltmeter and cross-checked post experiment (Extended Data Fig. 5a). In certain scenarios, we continually monitored electric currents in the circuit using a digital multimeter (Siglent, SDM3045), ensuring that they remained consistent (ranging from 5 to 8 μA) throughout the experiment (Extended Data Fig. 5b).

Differential interference contrast, RFP or GFP time-lapse images were acquired in an inverted epifluorescence microscope (Zeiss, Observer Z1) under a ×40 oil immersion objective, using a Retiga R6 (QImaging) scientific CCD camera and MetaMorph software (Molecular Devices), every second for up to 4 min. To simultaneously capture both green and red fluorescence signals, images were captured through a dual-band filter (76 HE) and a Zeiss colour camera (Axiocam 305).

### Image processing and data analysis

Time-lapse images were imported into ImageJ v.1.53m. Bacterial tracks (in a 6 s continuous time course) were marked by using the MtrackJ tool and plotted by using the Chemotaxis and Migration tool v.2.0 (Ibidi), as described^65^. To quantify directionality, we used directedness defined as the cosine of angle *θ* (cos*θ*), where *θ* is the angle between the endpoint of the cell’s trajectory and the vector of the applied electrical field. Averaged directedness (<cos*θ*>) values near −1 or 1 indicate robust directional migration towards the anode or cathode, respectively; values around 0 indicate random migration. Migration speed was calculated using the distance divided by the time that a cell migrated. Box graphs were generated using a standard R script. In some cases, galvanotaxis experiments and subsequent quantifications were assigned in a double-blinded manner.

### Immunostaining *S*. Typhimurium and quantification of flagellar orientation

During electrical field application, we passed the glass chamber containing the *S*. Typhimurium through a Benson flame multiple times. Given the small volume (usually less than 20 μl) inside the fluidic chamber, this quick heating effectively fixed the cells on a coverslip, preserving the flagellar orientation. The field orientation was marked on the slides. *S*. Typhimurium flagella were detected using a polyclonal antibody specific for *Salmonella* O- and H-antigens (Supplementary Table 1), followed by staining with an Alexa Fluor 555-conjugated secondary antibody. Fluorescence images were captured using a Carl Zeiss Observer Z1 inverted microscope equipped with a ×63 oil immersion objective lens and a Retiga R6 (QImaging) scientific CCD camera. The images were then imported into ImageJ. Flagella were marked as straight lines, and their orientations were measured by vector *θ* and calculated as cosine *θ*, where *θ* represents the angle between the flagellar line with respect to the applied electrical field vector, or to the horizon for the no electrical field controls. Rose plots representing the distribution of *θ* across 12 angle intervals and their abundance in percentage were generated using a standard script in MATLAB (Mathworks).

### Ex vivo bacterial tropism assay

In our study, we employed a well-characterized *E. coli* (derived from a commensal strain of *E. coli* K12) constitutively expressing dTomato, and a pathogenic derivative of *S*. Typhimurium 14028S constitutively expressing GFP, utilizing an ex vivo caecum model^25^. Freshly dissected caecum tissues were mounted in mouse Ringer’s solution within Petri dishes and challenged with a mixture of *E. coli* and *S*. Typhimurium at ratios of 20:1 or 1:1, with a concentration of 10^8^ c.f.u.s ml^−1^. The actual c.f.u.s were verified through serial dilutions and plate counts. Fluorescence and bright-field images were captured at the outset and 30 min after gentle washing using a ZEISS SteREO Discovery.V12 fluorescence microscope equipped with RFP and GFP filters and a Zeiss Axiocam camera.

In select experiments, fluorescently labelled microspheres (Invitrogen, F8814, 1.0 μm, excitation/emission: 365/415 nm) were also included at a 1:2 ratio of beads to bacteria. Fluorescence images were taken using an upright confocal microscope (Zeiss LSM 900) with a ×5 lens, enabling comprehensive scanning of entire Peyer’s patches and surrounding villus epithelium within ~1 h post incubation and washes. Image acquisition utilized AxioVision or Zen software (Carl Zeiss), with further processing in ImageJ.

The readily identifiable FAE regions, along with adjacent villi matched in size to the FAEs and villi located away from the Peyer’s patch, were designated as ROIs in the bright-field images. These ROIs were then aligned with the fluorescence images to study the spatial distribution of *S*. Typhimurium/*E.coli*/Beads. The spatial distribution and quantification of the latex beads were determined as a percentage of each ROI area by thresholding the bead fluorescence signal, taking advantage of their consistent brightness and uniformity. The bacterial affinity towards FAE or villus epithelium, as defined by the ROIs, was evaluated using normalized mean fluorescence intensities or ratios of fluorescence intensities for relative quantitation and comparison.

For visualization, representative high-resolution images shown in Extended Data Fig. 9b,c were obtained with a ×20 lens, primarily to illustrate the distribution patterns of bacteria and beads, and were not subjected to quantitative analysis.

### Competitive epithelium targeting assay in an ex vivo caecum model

The competitive assays were performed as previously described^11,49^ but executed in our ex vivo setup. Groups of female C57BL/6J (The Jackson Laboratory) mouse caeca (*n* = 4 mice in each condition) were mounted on PDMS gel in custom-made chambers as described in the ‘Animals and surgery’ section. The day before the experiment, bacterial cells were grown overnight under aerobic conditions and resuspended in mouse Ringer’s solution at a density of 10^9^ c.f.u.s ml^−1^. Caeca mounted on a PDMS gel with its lumina facing up were incubated with a 1:1 mixture containing 10^8^ c.f.u.s ml^−1^ of each bacterial strain in 5 ml mouse Ringer’s solution. Approximately 30 min after incubation at 37 °C in the dark, the tissues were gently washed 3× with mouse Ringer’s solution to remove floating bacteria. From each caecum, FAE inside Peyer’s patch or villus epithelium away from the Peyer’s patch were collected under a dissecting microscope (Zeiss, Stemi 508) using a 2 mm biopsy punch (Miltex, Integra 3331). We removed excess wash buffers to minimize contamination of residual bacteria before punching the tissues. Given the short incubation time and our interest in the bacteria adhering to the epithelia (Figs. 1d–f and 6b), we dislodged most of the epithelium-associated bacteria by vigorously pipetting the tissue with 1 ml tips into 500 μl of PBS in Eppendorf tubes. Bacteria were enumerated by spreading serial 6-fold dilutions of each sample on LB plates containing the appropriate antibiotics (Supplementary Table 1). This enabled us to determine the c.f.u.s of each strain per unit of epithelial area (mm^2^). The competitive index was calculated using the formula: log(c.f.u.s of strain 2/c.f.u.s of strain 1)^11,49^ (Extended Data Fig. 8b). Data analysis and graphing were performed using GraphPad Prism v.10 for Windows.

### Statistical analysis

No statistical methods were used to predetermine sample sizes, but our sample sizes are similar to those reported in previous publications^11^. Data were collected from at least two independently repeated experiments, unless otherwise indicated in the figure legends. No animals or data points were excluded from the analyses. Scientists were not blinded for the assignment of the experiments and data analysis except in some cases; galvanotaxis experiments and subsequent quantifications were assigned in a double-blinded manner. Data distribution was assumed to be normal, but this was not formally tested. Unpaired, two-tailed Student’s *t*-test and one-way analysis of variance (ANOVA) followed by post hoc Tukey HSD test was used for comparisons among two groups or multiple groups (more than two), respectively. Statistical analysis was performed using either Microsoft Excel or GraphPad Prism v.10 for Windows. *P < 0.05, **P < 0.01.

### Reporting summary

Further information on research design is available in the Nature Portfolio Reporting Summary linked to this article.

## Data availability

Raw data can be found in the source data file for each figure item. Any additional data can be requested from the corresponding authors. Source data are provided with this paper.

## Code availability

No custom code was used to analyse any of the data in this paper.

## Extended Data

**Extended Data Fig. 6 |:**
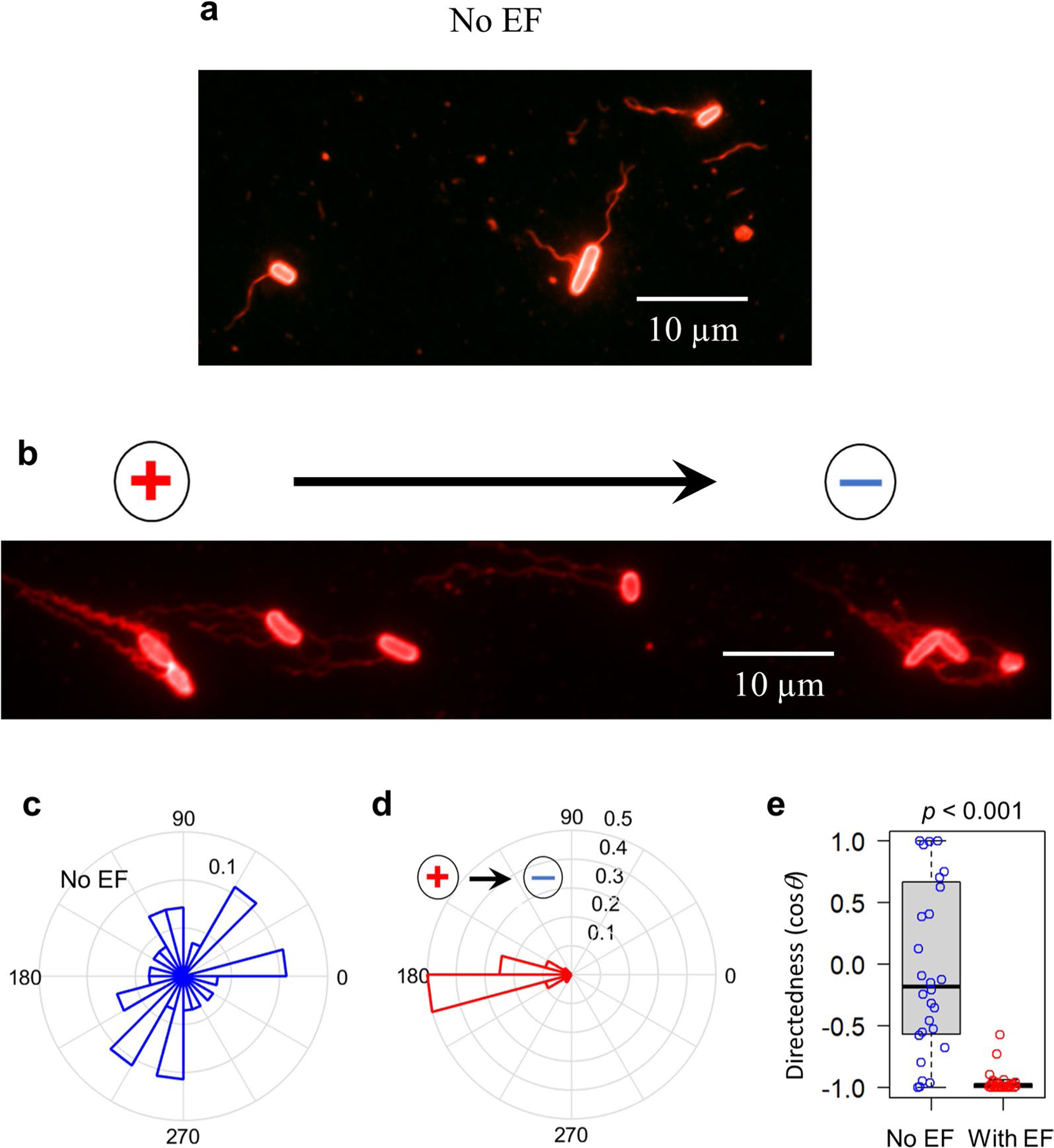
Electrical field induces flagellar redistribution exclusively at the rear of the *S*. Typhimurium body at the anode side. **a**, A fluorescence image showing flagella arranged in various directions in the absence of an electrical field (No EF). Scale bar, 10 μm. **b**, A fluorescence image demonstrating flagellar redistribution to the rear of the cell body on the anode side when an electrical field is present in the indicated orientation. Scale bar, 10 μm. **c**, A rose plot illustrating the random arrangement of flagella in the absence of an electrical field (No EF, n = 28 cells). The ring unit represents frequency (percentage). **d**, A rose plot revealing that an electrical field in the indicated orientation enforces flagella to redistribute at the rear of the cell body, facing the anode side at an angle less than 60 degrees (n = 32 cells). The ring unit represents frequency (percentage). **e**, Quantification of flagellar orientation as cos*θ*, where *θ* are the angles in (c) and (d). Negative values indicate orientation towards the anode (left), and positive values indicate orientation towards the cathode (right). Each circle represents an individual cell (n = 28 and 32 respectively). *p* < 0.001 by unpaired, two-tailed Student’s t-test. Box tops indicate the 75th percentile, box bottoms indicate the 25th percentile, center lines indicate the median, and whiskers indicate maximum and minimum values.

**Extended Data Fig. 7 |:**
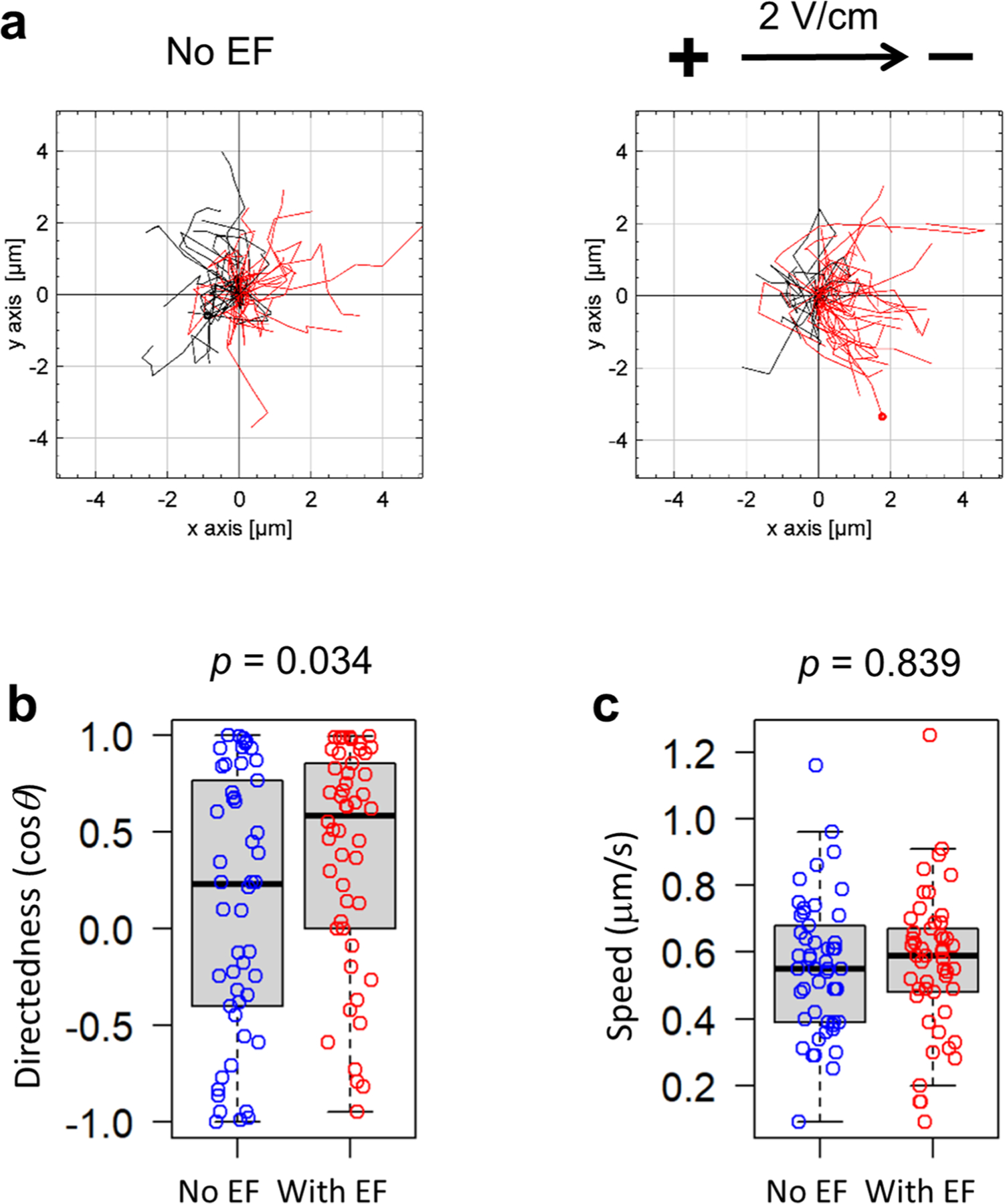
*B. subtilis* do not undergo robust galvanotaxis *in vitro.* **a**, Migration trajectories of *B. subtilis* over 6 seconds in the absence (No EF) or presence of an electrical field (2 V/cm) with the field polarity as shown. Red: to the right or cathode, black: to the left or anode. **b**, Quantification of directionality in cos*θ*. Each circle represents an individual cell (n = 50), *p* = 0.034 by unpaired, two-tailed Student’s *t*-test. **c**, Quantification of migration speed (μm s^−1^). Each circle represents an individual cell (n = 50), *p* = 0.839 by unpaired, two-tailed Student’s *t*-test. For panels (**b**) and (**c**) box tops indicate the 75th percentile, box bottoms indicate the 25th percentile, centre lines indicate median, and whiskers indicate maximum and minimum.

**Extended Data Fig. 8 |:**
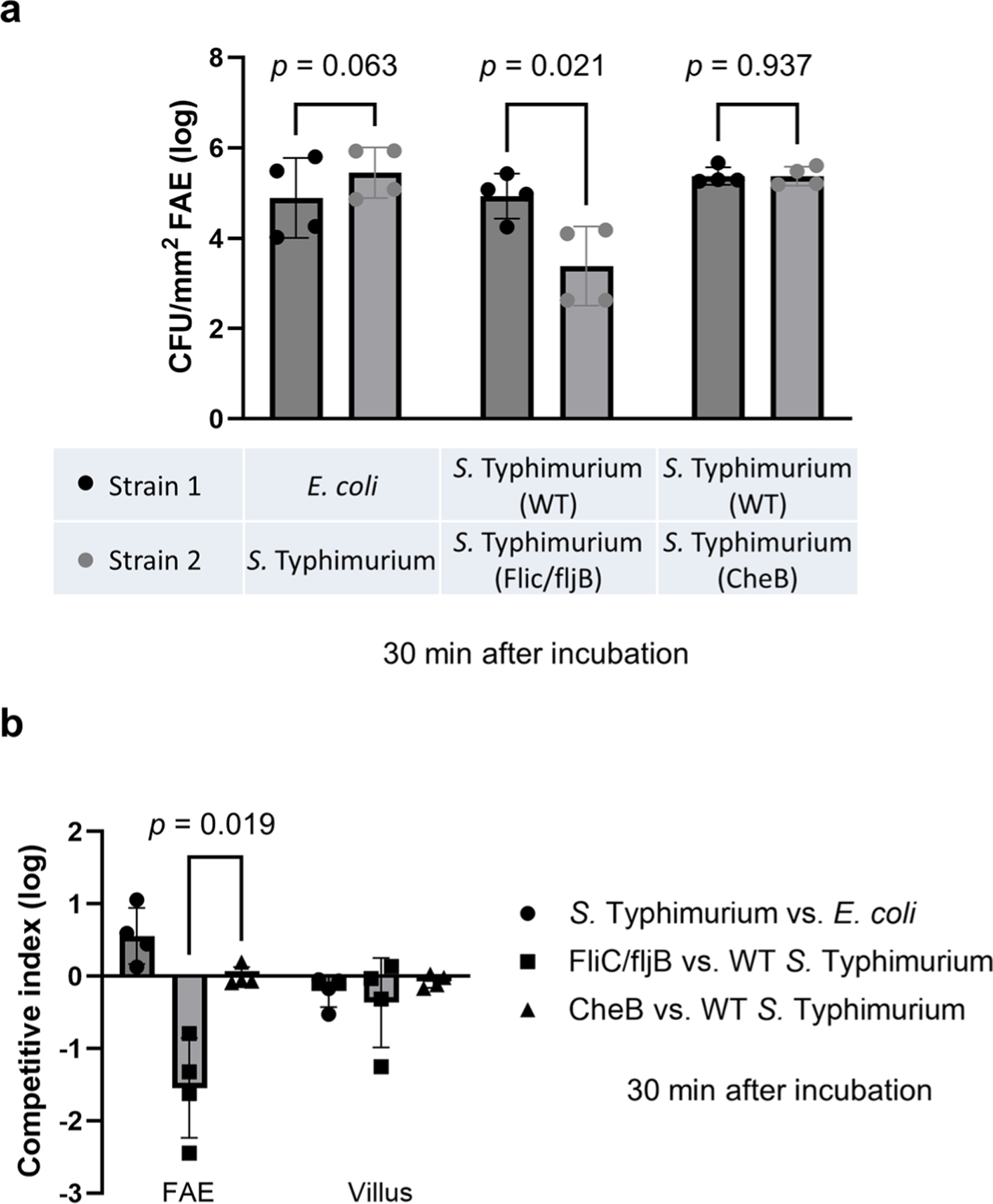
Flagella-dependent targeting to FAE is chemotaxis-independent. Mouse caeca were incubated with the indicated *E. coli* and/or *S*. Typhimurium strain or stain mixtures *ex vivo*, and epithelia (FAE or Villus) were collected by 2 mm biopsy punches 30 min after inoculation. **a**, Bars represent geometric means ± standard errors of the indicated bacterial c.f.u.s recovered from the FAEs in log10 (*n* = 4 mice in each condition). *P* values were calculated by unpaired, two-tailed Student’s *t*-test. **b**, Bars represent geometric means ± standard errors of the competitive index of the recovered bacteria from each epithelium (FAE or Villus) in log10 (*n* = 4 mice in each competition pair). *p* = 0.019 by unpaired, two-tailed Student’s *t*-test.

**Extended Data Fig. 9 |:**
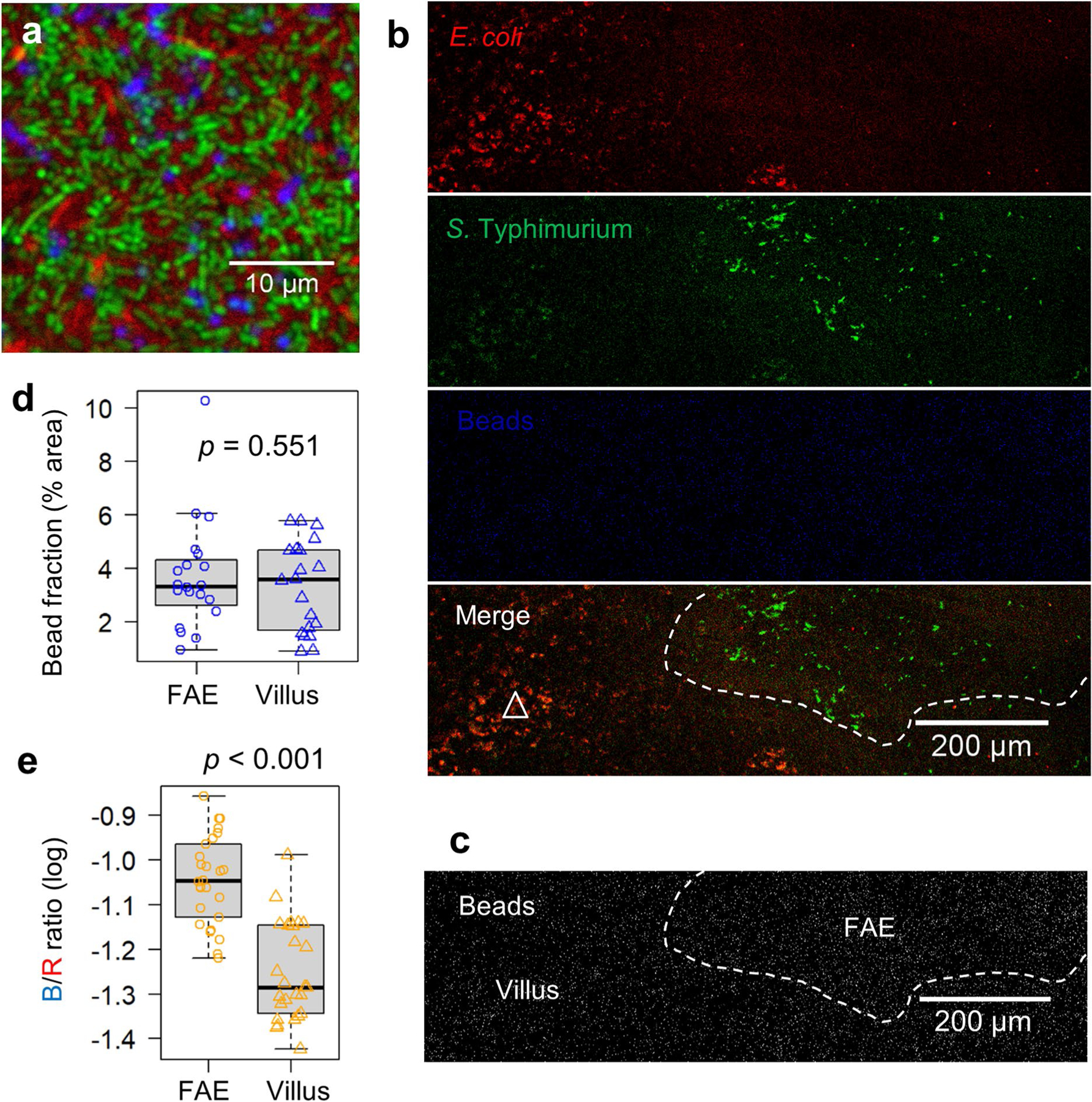
Differential recruitment of latex beads and bacteria to FAE and villus epithelium. **a**, A confocal microscopy image displaying the initial inoculum mixture containing *E. coli* K12 (red), *S*. Typhimurium 14028S (green), and latex bead (blue) at a 1:1:1 ratio (10^8^ c.f.u.s ml^−1^ in mouse Ringer’s solution). **b**, Representative confocal microscopy image of mouse caecum epithelium 30 min post-incubation with the tagged bacteria and bead mixture. The white dashed line delineates the FAE from the surrounding villus epithelium (indicated by a white triangle). Scale bar, 200 μm. **c**, Enhanced contrast image of the blue fluorescence channel from (**b**), illustrating the homogeneous distribution of latex beads across both the FAE and adjacent villus epithelium, demarcated by a dashed line. Scale bar, 200 μm. **d**, Quantification of fluorescent bead fraction within the FAE and villus epithelium (% area). Analysis was based on observations from multiple FAEs and corresponding villus regions across two independent experiments (n = 4 mice). *p* = 0.551 by unpaired two-tailed Student’s *t*-test. **e**, Analysis of mean Blue (beads) vs. Red (*E. coli*) fluorescence intensity (B/R) ratios associated with the FAE and villus epithelium, presented in log10 scale. Calculations were derived from multiple FAEs and matched villus regions over two repeated experiments (n = 4 mice). *p* < 0.001 by unpaired, two-tailed Student’s *t*-test, indicating a significant difference in the distribution of *E. coli* K12 between the two epithelial types. For panels (**d**) and (**e**) box tops indicate the 75th percentile, box bottoms indicate the 25th percentile, centre lines indicate median, and whiskers indicate maximum and minimum.

**Extended Data Fig. 10 |:**
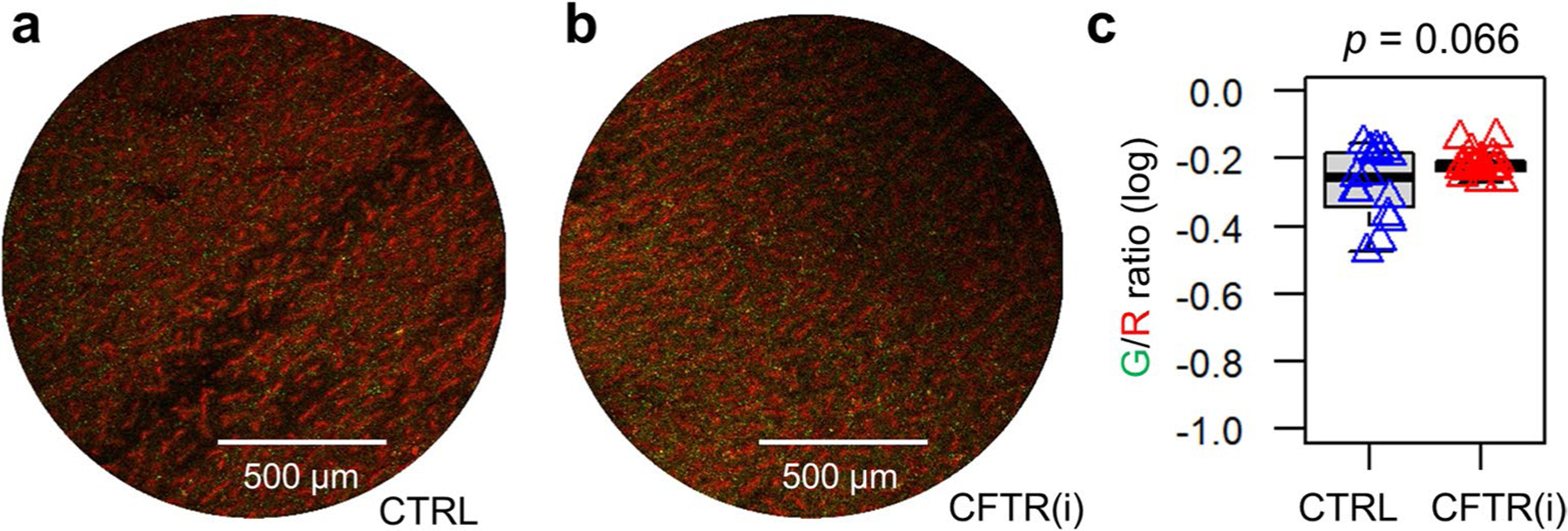
CFTR inhibition does not change *S*. Typhimurium and *E. coli* recruitment in villi away from FAE. **a**, A representative confocal image shows *E. coli* (red) associating with villi and sparsely scatted *S*. Typhimurium (green). Bar, 500 μm. **b**, A representative confocal image displays a similar distribution pattern of *E. coli* (red) and *S*. Typhimurium (green) in the villus epithelium in the presence of CFTR inhibitor (CFTR (i)) compared to the absence of CFTR (CTRL) in (**a**). **c**, Quantification of mean Green/Red fluorescence intensity ratios associated with the vast villi away from FAE in the absence or presence of CFTR(i). Data in common logarithm were calculated from multiple regions of two repeated experiments in each condition (*n* = 4 mice). *p* = 0.066 by unpaired, two-tailed Student’s t-test. Box tops indicate the 75th percentile, box bottoms indicate the 25th percentile, centre lines indicate median, and whiskers indicate maximum and minimum.

## Supplementary Material

Video1

Video2

Video3

Video4

Video5

Video6

Video7

RawData
