# Supplementary material for: Gut epithelial electrical cues drive differential localization of enterobacteria
Source: Nat Microbiol. 2024 Aug 20;9(10):2653–65. doi: 10.1038/s41564-024-01778-8 (PMC11445056; doi:10.1038/s41564-024-01778-8)
Supplement: Supplementary file 1 [file 41564_2024_1778_MOESM1_ESM.pdf]

nature portfolio

Corresponding author(s): Yaohui Sun, Alex Mogilner, Min Zhao

Last updated by author(s): June 18, 2024

Reporting Summary

Nature Portfolio wishes to improve the reproducibility of the work that we publish. This form provides structure for consistency and transparency in reporting. For further information on Nature Portfolio policies, see our Editorial Policies and the Editorial Policy Checklist.

Statistics

For all statistical analyses, confirm that the following items are present in the figure legend, table legend, main text, or Methods section.

n/a	Confirmed	
☐	☒	The exact sample size (*n*) for each experimental group/condition, given as a discrete number and unit of measurement
☐	☒	A statement on whether measurements were taken from distinct samples or whether the same sample was measured repeatedly
☐	☒	The statistical test(s) used AND whether they are one- or two-sided<br>*Only common tests should be described solely by name; describe more complex techniques in the Methods section.*
☒	☐	A description of all covariates tested
☒	☐	A description of any assumptions or corrections, such as tests of normality and adjustment for multiple comparisons
☐	☒	A full description of the statistical parameters including central tendency (e.g. means) or other basic estimates (e.g. regression coefficient) AND variation (e.g. standard deviation) or associated estimates of uncertainty (e.g. confidence intervals)
☐	☒	For null hypothesis testing, the test statistic (e.g. *F*, *t*, *r*) with confidence intervals, effect sizes, degrees of freedom and *P* value noted<br>*Give P values as exact values whenever suitable.*
☒	☐	For Bayesian analysis, information on the choice of priors and Markov chain Monte Carlo settings
☒	☐	For hierarchical and complex designs, identification of the appropriate level for tests and full reporting of outcomes
☒	☐	Estimates of effect sizes (e.g. Cohen's *d*, Pearson's *r*), indicating how they were calculated

Our web collection on statistics for biologists contains articles on many of the points above.

Software and code

Policy information about availability of computer code

Data collection	WinWCP V4 (Strathclyde Electrophysiology Software), Axiovision 4.8.2 (Carl Zeiss), Zen 3.7 (Carl Zeiss), pClamp 10 (Molecular Devices), MetaMorph 7.7.4 (Molecular Devices).
Data analysis	Excel (Microsoft 365), ImageJ 1.53h (NIH), MATLAB 2022 (MatchWorks), R 4.2.2 (r-project), Prism 10.2.0 (GraphPad).

For manuscripts utilizing custom algorithms or software that are central to the research but not yet described in published literature, software must be made available to editors and reviewers. We strongly encourage code deposition in a community repository (e.g. GitHub). See the Nature Portfolio guidelines for submitting code & software for further information.

Data

Policy information about availability of data

All manuscripts must include a data availability statement. This statement should provide the following information, where applicable:

- Accession codes, unique identifiers, or web links for publicly available datasets
- A description of any restrictions on data availability
- For clinical datasets or third party data, please ensure that the statement adheres to our policy

Source Data are available for Figs. 1 to 6 and Extended Data Figs. 1, 2, 4, 5, 6, 7, 8, 9 and 10.

Research involving human participants, their data, or biological material

Policy information about studies with human participants or human data. See also policy information about sex, gender (identity/presentation), and sexual orientation and race, ethnicity and racism.

Reporting on sex and gender	N/A
Reporting on race, ethnicity, or other socially relevant groupings	N/A
Population characteristics	N/A
Recruitment	N/A
Ethics oversight	N/A

Note that full information on the approval of the study protocol must also be provided in the manuscript.

Field-specific reporting

Please select the one below that is the best fit for your research. If you are not sure, read the appropriate sections before making your selection.

☒ Life sciences ☐ Behavioural & social sciences ☐ Ecological, evolutionary & environmental sciences

For a reference copy of the document with all sections, see nature.com/documents/nr-reporting-summary-flat.pdf

Life sciences study design

All studies must disclose on these points even when the disclosure is negative.

Sample size	At least 4 mice were used in each group. Sample size was chosen according to institutional directives and in accordance with the 3Rs rules (Replacement, Reduction and Refinement) guiding principles underpinning the humane use of animals in search, but not statistical analyses were performed to predetermine the sample sizes.
Data exclusions	No data were excluded from the analysis.
Replication	All the experiments were performed at least twice, with all attempts at data replication being successful.
Randomization	Mice were randomly allocated to different treatments.
Blinding	In some cases, galvanotaxis experiments and subsequent quantifications were assigned in a double-blinded manner.

Reporting for specific materials, systems and methods

We require information from authors about some types of materials, experimental systems and methods used in many studies. Here, indicate whether each material, system or method listed is relevant to your study. If you are not sure if a list item applies to your research, read the appropriate section before selecting a response.

Materials & experimental systems

n/a	Involved in the study	
☐	☒	Antibodies
☒	☐	Eukaryotic cell lines
☒	☐	Palaeontology and archaeology
☐	☒	Animals and other organisms
☒	☐	Clinical data
☒	☐	Dual use research of concern
☒	☐	Plants

Methods

n/a	Involved in the study	
☒	☐	ChIP-seq
☒	☐	Flow cytometry
☒	☐	MRI-based neuroimaging

Antibodies

Antibodies used	Rabbit Salmonella Polyclonal Antibody, MyBioSource, Catalog # MBS535017.

Validation	The Rabbit Salmonella Polyclonal Antibody was validated by the provider, which is posted in th vendor's website.

Animals and other research organisms

Policy information about studies involving animals; ARRIVE guidelines recommended for reporting animal research, and Sex and Gender in Research

Laboratory animals	6–10-week-old C57BL/6 mice were purchased from Jackson lab. 6–10-week-old Wistar rats were purchased from Charles River. Both male and female mice or rats were used for each experiment unless otherwise specified in the figure legend. The rodents were maintained under a strict 12-h light cycle and given a regular chow diet in a specific pathogen-free facility at the University of California (UC), Davis.
Wild animals	No wild animals were used.
Reporting on sex	6-10 week old mice and rats of both sexes were randomly assigned to experimental groups.
Field-collected samples	This study did not involve samples collected from the field.
Ethics oversight	All animal experiments were performed per regulatory guidelines and standard set by the institutional Animal Care and Use Committee of UC Davis.

Note that full information on the approval of the study protocol must also be provided in the manuscript.

Plants

Seed stocks	N/A
Novel plant genotypes	N/A
Authentication	N/A
