# Supplementary material for: Gut epithelial electrical cues drive differential localization of enterobacteria
Source: Nat Microbiol. 2024 Aug 20;9(10):2653–65. doi: 10.1038/s41564-024-01778-8 (PMC11445056; doi:10.1038/s41564-024-01778-8)
Supplement: Supplementary file 2 [file 41564_2024_1778_MOESM2_ESM.pdf]

Supplementary Table 1: Special reagents, plasmids, and bacteria used in this study

	Description and/or relevant information	Reference/Source
**Reagents**		
Blue beads	1.0 μm FluoSpheres™ Carboxylate-Modified Microspheres. Excitation/Emission: 365/415 nm	Invitrogen, F8814
DIDS	4,4′-Diisothiocyano-2,2′-stilbenedisulfonic acid (DIDS), general anion exchange inhibitor	Sigma, D3514
AMIL	Amiloride, a general sodium channel (ENaC) blocker	Sigma, A7410
CFTR(inh)-172	5-[(4-Carboxyphenyl)methylene]-2-thioxo-3-[(3-trifluoromethyl)phenyl-4-thiazolidinone, a specific CFTR inhibitor	Sigma, C2992
$DiBAC_4(3)$	Bis (1,3-dibutylbarbituric acid) trimethine oxonol, a translational membrane potential dye	Sigma, D8189
**Antibodies**		
Salmonella antibody	*Salmonella*, Polyclonal Antibody	Mybiosource, MBS535017
2nd antibody	Alexa Fluor™ 555, goat anti rabbit IgG (H+L)	Invitrogen, A21429
**Plasmids**		
pFT/RalFc	pBBR1-MCS4 based low-copy number plasmid expressing FT::RalF(350-374). CmR.	[25]
pdTFT/RalFc	In pFT/RalFc, constitutively expressing dTomato	This work
pGFT/RalFc	In pFT/RalFc expressing GFP mut3 under *aphA3* promoter. CmR.	[25]
**Bacteria**		
DH5α	A laboratory strain derived from *E. coli* K12	
B. subtilis	A laboratory *Bacillus subtilis* strain	ATCC 6051
Red EC	DH5α, + pdTFT/RalFc, CmR	This work
IR715	Wild-type *Salmonella enterica* subsp. *enterica* serovar Typhimurium, ATCC 14028S, NalR derivative	[64]
Green ST	IR715, + pGFT/RalFc, NalR, CmR	This work
KLL14	IR715, glmS::mCherry, NalR, CmR	[25]
SW473	IR715, ∆fliC, fljB::MudJ, no flagellar, NalR, KanR	[43]
Green Flagella	SW473, + pGFT/RalFc, KanR, CmR	This work
CheB	ΔcheB::Tn10, TetR	[11]
Green CheB	CheB, + pGFT/RalFc, TetR, CmR	This work
